# Assessment of algorithmic performance in PET-CT segmentation of solitary lung tumors

**DOI:** 10.1371/journal.pone.0360849

**Published:** 2026-11-05

**Authors:** Duc Trung Le, Dániel Szabó, Sándor Aranyi, Márton Mikó, Zita Képes, Iván Mihovk, Anna Rebeka Kovács, Iván Gábor Nagy, Bence Farkas, István Hajdú, György Trencsényi, Lajos Trón, Miklós Emri, Gábor Opposits

**Affiliations:** 1 Division of Nuclear Medicine and Translational Imaging, Department of Medical Imaging, Faculty of Medicine, University of Debrecen, Debrecen, Hungary; 2 Scanomed Ltd., Debrecen, Hungary; Fondazione Policlinico Universitario Agostino Gemelli IRCCS, ITALY

## Abstract

**Background:**

PET-CT (Positron emission tomography–computed tomography), a nuclear medicine imaging technique is preferably used for lung tumor detection. However, manual image analysis may be time-consuming. To enhance efficiency, many automated segmentation algorithms have been developed. This study aims to develop a radiomics agreement-based method to evaluate segmentation algorithms and to compare the performance of two deep learning-based algorithms nnU-Net (No-new-net Convolutional Neural Network) and RECOMIA (Research Consortium for Medical Image Analysis Lung Cancer “Lung Cancer FDG-PET/CT v1.5”) for PET-CT solitary lung tumor segmentation.

**Methods and results:**

PET-CT images of 221 patients at the University of Debrecen’s Hospital were collected and anonymized. The predictions of segmented lung tumors done by nnU-Net and RECOMIA were compared with the manual segmented masks via Wilcoxon signed-rank test and Pearson correlation values, based on eight groups of radiomics parameters calculated by PyRadiomics software. The combined p-values were calculated using Brown’s method, which were 0.481 and < 0.001 for nnU-Net and RECOMIA, respectively. The absolute values of correlation were summed, which were 37.259 and 16.358 for nnU-Net and RECOMIA, respectively. Altogether, RECOMIA shows statistically greater differences, therefore, it performs worse than nnU-Net does. Statistical analyses with Intraclass correlation coefficients (ICC), Multivariate analysis of variance (MANOVA) and follow up pairwise t-test further confirmed stronger agreement between extracted radiomics features from nnU-Net predicted masks and manual masks than RECOMIA and manual masks, which validated the proposed comparison method.

**Conclusion:**

The superior performance of nnU-Net highlights the importance of training data alignment and model adaptability in achieving accurate and reliable segmentation in medical imaging. However, nnU-Net requirements for training and extra computational demands may limit its application in some clinical environments. Despite lower accuracy, RECOMIA remains valuable because of its efficiency and intuitiveness. These findings provide valuable guidance for selecting segmentation algorithms in real-world clinical and research settings.

## Introduction

Lung cancer remains the leading cause of cancer-related death worldwide, accounting for the highest mortality rates among both men and women [1]. Early and accurate diagnosis plays a crucial role in improving patient outcomes [2]. Because of its advantages in the integration of metabolic and anatomical information, Positron emission tomography-computed tomography (PET-CT) is one of the most commonly used imaging modalities in the diagnosis, staging and monitoring of lung cancer [3,4]. However, accurate diagnosis requires careful examination of PET-CT images by a skilled nuclear medicine expert, although routinely performed in clinical practice, remains time-consuming and labor-intensive.

Recently, a large number of automated segmentation algorithms have been developed to increase the efficiency and consistency of this process [5,6]. These algorithms use advanced artificial intelligence techniques including deep learning and machine learning to process and analyze images. The performance of these algorithms is extremely significant, as precise segmentation and diagnosis can impact the subsequent steps of the healthcare workflow, such as treatment planning, response assessment and most importantly, patient outcomes [7].

Radiomics involves the application of advanced mathematical and statistical methods to extract numerous quantitative features, which are called radiomics parameters from standard clinical images (e.g., computed tomography (CT), magnetic resonance imaging (MRI) and positron emission tomography (PET)). These parameters are believed to contain valuable information regarding tissue and lesion characteristics such as heterogeneity, shape and spatial texture, which might be undetectable by the naked eye [8–10]. Unlike traditional biopsies, which sample only a small portion of the tumor, radiomics features can offer a comprehensive characterization of the entire tumor, which is usually heterogeneous. This provides a deeper understanding of tumor biology and after rigorous validation, radiomics features could be considered as clinically relevant non-invasive prognostic or predictive biomarkers [8,11,12]. Therefore, these features could significantly improve diagnosis, treatment response and survival outcomes [11].

Several studies have compared PET-CT segmentation methods using conventional overlap- and volume-based metrics such as Dice similarity coefficient (DSC), Hausdorff distance (HD) and Mean surface distance (MSD) [13–15]. Although these metrics are useful for assessing spatial agreement between segmented masks, they may not adequately reflect the clinically relevant characteristics of the delineated tumor, such as tumor shape, intensity, and texture characteristics [16]. Until now, limited published literature has investigated whether agreement in radiomics-derived quantitative features can be used to compare the segmentation algorithms. Therefore, the aim of the study is to develop a radiomics agreement-based method for evaluating performance of segmentation algorithms. Specifically, we applied this method to compare the performance of two deep learning-based algorithms: nnU-Net (No-new-net Convolutional Neural Network) [17] and RECOMIA (Research Consortium for Medical Image Analysis “Lung Cancer FDG-PET/CT v1.5”) [18] for PET-CT solitary lung tumor segmentation. In this study, radiomics analysis was not used for tumor segmentation itself, but rather as a quantitative assessment tool applied on the output segmented masks, to evaluate the ability to represent tumor characteristics. By evaluating their performance, we attempt to assist the future development of medical image processing technology, specifically selection for specific clinical and research needs. Through this research, we also hope to contribute a small effort to enhance the accuracy and efficiency of lung tumor segmentation, to improve patient care and outcomes.

## Materials and methods

### Study design and Ethical approval

This retrospective methodological study was conducted using 221 previously acquired PET-CT images of patients with lung tumors at University of Debrecen Hospital. The study was approved by University of Debrecen Ethics Committee (Protocol ID (DE RKEB/IKEB-nél) 6504−2023, 13^th^ September 2023), in accordance with the relevant guidelines and regulations. Written informed consent was obtained from the patients, to participate in the study.

### Datasets

PET/CT scans were obtained using integrated Philips Vereos PET/CT system (Philips Medical Systems, Cleveland, USA). Patients fasted for at least 6 hours prior to the injection of 3 MBq/kg (maximum of 520 Mbq) of [^18^F]FDG, and image acquisition started 60 min post-injection. Images were acquired at 60 (BMI < 25); 75 (BMI = 25–30); 90 (BMI = 30–40); 120 (BMI > 40) sec per bed position (axial field of view 160 mm, overlap 30%). PET images were reconstructed using an ordered subset expectation maximization (OSEM) or 3D row-action maximum likelihood algorithm (3D-RAMLA) algorithms (288 x 288 matrix; 2 mm slice thickness); attenuation and scatter correction were applied by CT images. CT images were accomplished using low-dose protocols (120 kV tube voltage, IDOSE dose optimization, 64 slices), with filtered back projection (FBP) reconstruction technique (3 mm slice thickness). The PET-CT images of 221 patients with lung cancer at the University of Debrecen’s Hospital were downloaded from the eRAD server (version: ERAD_60) and anonymized using the DICOM ToolKit version: DCMTK-3.5.4 (Digital Imaging and Communications in Medicine Toolkit) package [19,20].

### Pre-processing

By using the “in-house” developed image processing toolkit dcm2mnc and mnc2nii, we converted PET-CT images from DICOM (Digital Imaging and Communications in Medicine) to MINC (Medical Imaging NetCDF) and then to NIfTI (Neuroimaging Informatics Technology Initiative) format, which is accepted by the nnU-Net algorithm. Image resizing was also performed to match the dimensions of the PET and CT images.

### Algorithms

The nnU-Net algorithm was developed by the Applied Computer Vision Lab (ACVL) of Helmholtz Imaging and the Division of Medical Image Computing at the German Cancer Research Center (DKFZ). It is a deep learning image analysis method based on a convolutional neural network (CNN). It can adapt to provided training cases and automatically configure a segmentation pipeline without manual intervention. To ensure robustness, up to three configurations are trained using a five-fold cross-validation. The configuration with the best validation performance based on Dice similarity coefficient (DSC) is then selected to generate the final fully trained models [17]. nnU-Net model 535 was trained on 178 PET-CT images and their corresponding expert-approved masks via five-fold cross-validation. These expert-approved masks were first generated by 7 algorithms: Fuzzy C-means, K-Means, Gaussian mixture model, Active contour, Region growing algorithm, Random walker and Majority voting method of the former ones. A panel of five nuclear medicine specialists graded these masks on a three-point scale, and the highest-quality masks were selected for training. Each nnU-Net model 535 configuration from the five-fold cross-validation was also evaluated using the Jaccard index, where higher values indicate greater similarity to the reference mask (see Fig 1). nnU-Net model 535 was tested using an independent set of 43 PET-CT images, with PET and CT channels jointly used during segmentation inference and manual masks used as reference. These testing masks were delineated by one of the original nuclear medicine experts who participated in the grading process and one other nuclear medicine expert from University of Debrecen. The testing process of nnU-Net model 535 resulted in 43 predicted masks (see Fig 2). By contrast, nnU-Net models 502 and 503 were both trained and tested exclusively on the same 43 images that served as the independent test set for model 535. Model 502 was trained with 9 PET-CT images and tested on the remaining 34 images, producing 28 valid masks and 6 failed cases (no mask). Model 503 was trained with 21 PET-CT images and tested on the remaining 22, resulting in 14 valid masks but 8 failed cases (7 cases with no mask and 1 case with multiple masks) (see Fig 2). The allocations of PET-CT images for training, testing and model outputs are summarized in Table 1. Overall, model 535 produced the highest quality results, whereas 503 did not outperform 502. Therefore, nnU-Net model 535 was selected for all further analyses. (see Table 3 and Table 4 in section Statistical based accuracy of nnU-Net predictions in Results).

**Table 1:** Overview of PET-CT images allocations for training and testing of RECOMIA, nnU-Net model 535, 502 and 503 and model output (valid predicted masks).

Segmentation algorithm	RECOMIA	nnU-Net model 535	nnU-Net model 502	nnU-Net model 503
**Training dataset**	320	178	9	21
**Testing dataset**	43	43	34	22
**Valid predicted masks**	41	43	28	14
**Failed predicted masks**	2	0	6	8
**Type of failure**	No mask generated	No failure	No mask generated	No mask or multiple masks generated

**Fig 1:**
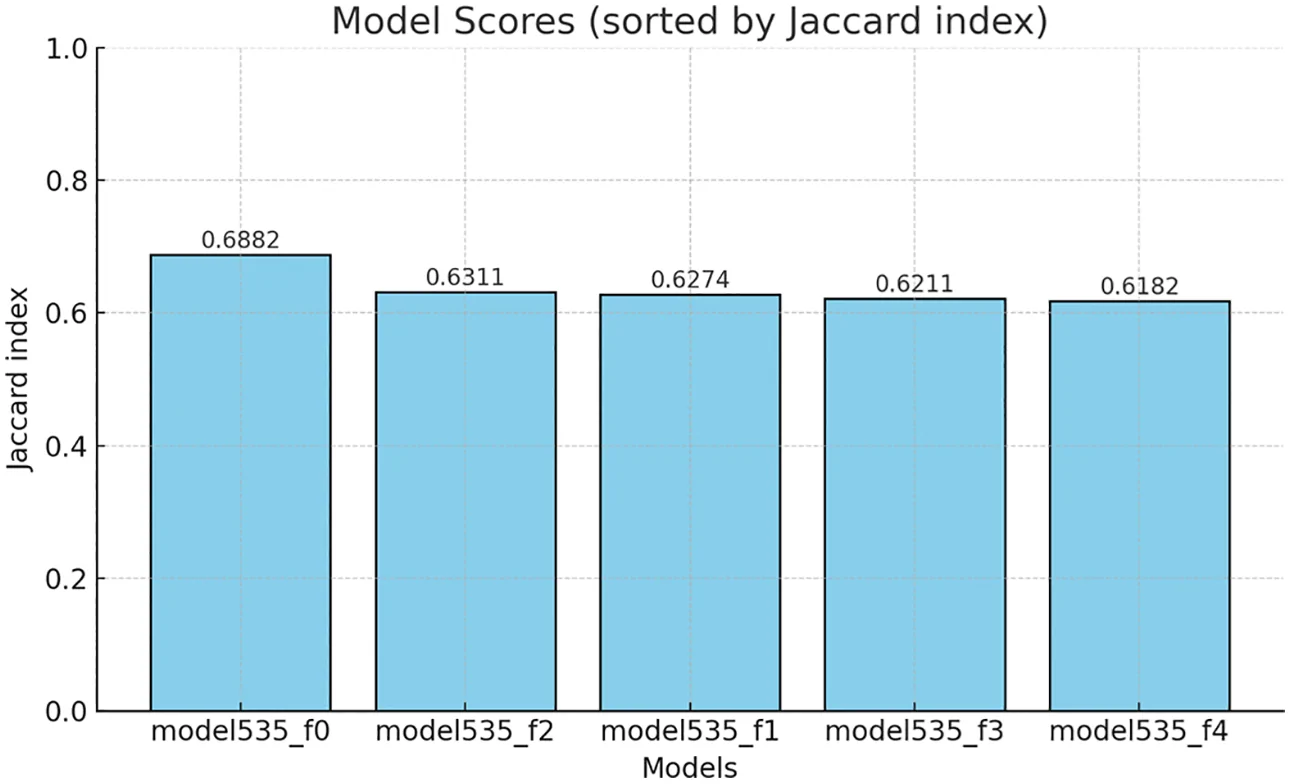
Five-fold Cross-validation performance for nnU-Net model 535 on the training set. The bars show the per-fold Jaccard index, sorted from highest to lowest.

**Fig 2:**
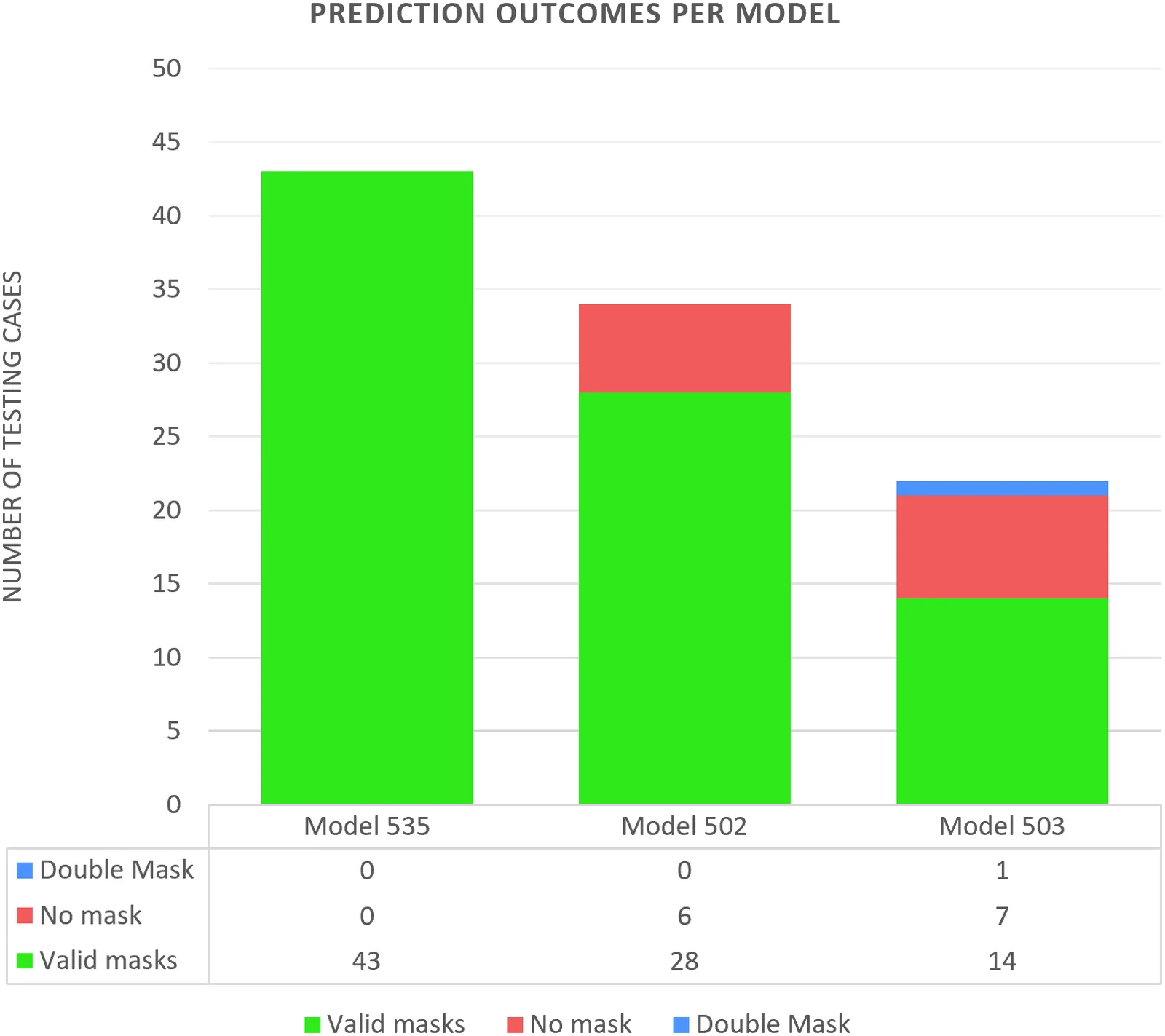
Prediction outcomes per nnU-Net model. The bars show the number of cases with valid predicted mask, no mask and double masks.

The RECOMIA algorithm was developed by the Research Consortium for Medical Image Analysis (RECOMIA), a not-for-profit organization with board members from Sahlgrenska University Hospital, Lund University, Chalmers Technical University and Skåne University Hospital. It is a deep-learning lung tumor segmentation algorithm, which is also based on convolutional neural network (CNN). This algorithm has already been pre-trained by RECOMIA using a dataset of 320 PET/CT studies manual masks from patients with suspected lung cancer [21]. PET/CT scans were obtained using integrated PET/CT systems (Siemens Biograph 64 Truepoint, Siemens Healthineers, Erlangen, Germany and GE Discovery MI, GE Healthcare, Chicago, USA). Patients fasted for at least 4 hours prior to the injection of 4 MBq/kg (maximum of 400 Mbq) of [^18^F]FDG, and image acquisition started 60 min post-injection. Images were acquired at 3 min or 1.5 min per bed position, at Sahlgrenska and Skåne University Hospital, respectively. Siemens PET images were reconstructed using an ordered subset expectation maximization (OSEM) 3D algorithm (four iterations, eight subsets; 168 x 168 matrix; 3 mm slice thickness). GE PET images were reconstructed using block-sequential regularized expectation maximization (BSREM) algorithm (Q.Clear, β = 550) with time-of-flight and point spread functions (256 × 256 matrix; 2.7 × 2.7 mm² pixel size; 2.8 mm slice thickness). CT images were acquired for attenuation correction and anatomical correlation using either low-dose (120 kV tube voltage) or diagnostic protocols (100 kV tube voltage), with adaptive statistical iterative reconstruction technique [22]. We uploaded 43 anonymized images in DICOM format to RECOMIA cloud-based platform, then removed any unnecessary files such as preview files or exam summary files. Lung tumor segmentations were performed using the version 1.5 implementation of the RECOMIA “Lung Cancer FDG-PET/CT” algorithm, resulting in 41 successfully generated segmentation masks, while the segmentation failed in 2 cases due to the absence of the valid prediction mask. The allocations of PET-CT images for training, testing and model outputs are summarized in Table 1. Since this study focused on solitary lung tumors, all secondary tumors detected were deleted from the predicted masks manually. We subsequently emailed the RECOMIA team, asking them to calculate the radiomics features for the 41 predicted masks.

### Radiomics

Following segmentation by nnU-Net algorithms, we extracted radiomics features using PyRadiomics software, which implements features definitions largely consistent with the image biomarker standardization initiative (IBSI) [10,23,24] using the predicted masks and corresponding PET images. The following configuration settings were used: binWidth: 5, normalize: true, normalizeScale: 100, resampledPixelSpacing: [1], interpolator: ‘sitkBSpline’, label: 1 and no filter was applied. In total, 107 radiomics features were obtained for each mask, which can be classified into eight groups, including first order, 3D shape, 2D shape, Gray Level Co-occurrence Matrix (GLCM), Gray Level Size Zone Matrix (GLSZM), Gray Level Run Length Matrix (GLRLM), Neighboring Gray Tone Difference Matrix (NGTDM) and Gray Level Dependence Matrix (GLDM). Similarly, we calculated the radiomics features using 43 manual masks, and asked RECOMIA team to calculate the radiomics features of 41 predicted masks generated by RECOMIA segmentation algorithm.

### Statistical analysis

This study aims to develop a radiomics agreement-based method to evaluate segmentation algorithms and to compare two deep learning-based algorithms nnU-Net and RECOMIA for PET-CT solitary lung tumor segmentation. The completed segmentation predictions were compared with the manual masks created by two nuclear medicine experts from the University of Debrecen. The comparison is based on eight groups of radiomics parameters calculated by the PyRadiomics software package [23]. The calculation of radiomics parameters and comparison methods are demonstrated in Fig 3. The radiomics features of nnU-Net and RECOMIA predicted masks were compared with manually segmented masks as the gold standard, via Wilcoxon signed-rank test [25] and Pearson correlation values in R software (version 4.1.0, R Core Team, Vienna, Austria). The normality of features was checked using Shapiro’s test [26]. We removed all parameters that didn't follow normal distribution. The combined p-values (calculated using Brown’s method [27]) and the aggregated absolute values of the correlations were subsequently used to compare the two segmentation methods. The significance level was set at 0.05.

**Fig 3:**
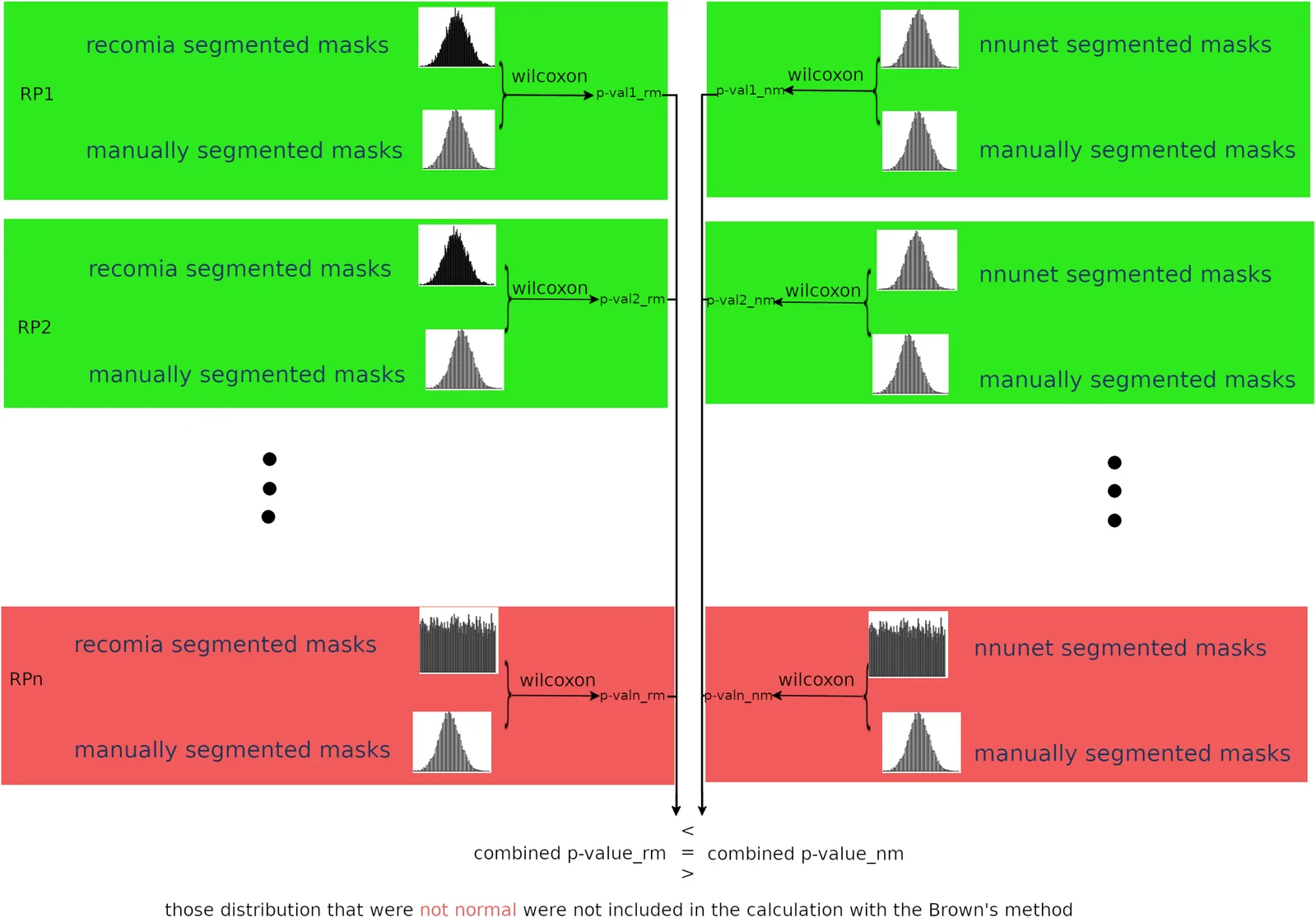
Workflow for radiomics parameters evaluation and comparison. For each radiomics parameter (RP1 … RPn), Wilcoxon signed rank tests were computed using prediction masks from nnU-Net, RECOMIA and manual masks (reference), yielding a p-value for RECOMIA (p-val_rm) and nnU-Net (p-val_nm). For features with normal distribution (green panel), we used Brown’s method to obtain an aggregate p-value for each algorithm. Features that failed the normality test were excluded from this step. Histograms represent feature distribution, RP = radiomics parameter, rm = RECOMIA vs manual and nm = nnU-Net vs manual.

We used Dice similarity coefficient (DSC), Hausdorff distance (HD), four similarity (Jaccard index, Pearson correlation coefficient, mutual information (MI), and normalized mutual information (NMI)) and two dissimilarity (Kullback-Leibler divergence (KLD), L1 norm, also known as Manhattan norm) to assess the accuracy of our nnU-Net models, since RECOMIA segmentation masks were not available [28–34]. Higher DSC suggests greater spatial overlap, whereas lower HD illustrates smaller boundary distance, which suggests closer agreement. Similarly, a high value of the similarity metric (Jaccard, Pearson, MI, NMI) indicates that the masks are similar, whereas a low value indicates that the two masks are not similar. The dissimilarity metrics (KLD, L1 norm) behave in the opposite direction, i.e., the higher the value, the greater the difference between the two masks. In summary, better segmentation performance is reflected by higher DSC and similarity values, together with lower HD and dissimilarity values.

To make the statistical basis of our proposed comparison approach more robust and well-established, we validated the proposed method using Intraclass correlation coefficient (ICC), which had already been used in literature for comparing segmentation algorithms [16,35], and Multivariate analysis of variance (MANOVA) analysis [36]. ICC was used to quantify the level of agreement between radiomics features extracted from segmentation outputs and manual reference masks. ICC values less than 0.5, between 0.5 and 0.75, between 0.75 and 0.9, and greater than 0.9 are associated with poor, moderate, good and excellent agreement, respectively [37]. Additionally, MANOVA was used to determine whether radiomics features differed significantly between segmentation methods. Statistically significant results were followed by pairwise t-test using Benjamini–Hochberg correction to determine the differences between each automated method and the manual reference mask.

### Robustness analysis

To assess the robustness of the results, a non-parametric bootstrap analysis [38] was performed with 1,000 iterations. For each iteration, the radiomics features extracted from nnU-Net model 535, RECOMIA and manual masks were resampled with replacement, Wilcoxon signed-rank test [25] and Pearson correlation values were recalculated for each feature. The normality of features was checked using Shapiro’s test [26] and all parameters that didn't follow normal distribution were removed. The combined p-values (calculated using Brown’s method [27]) and the aggregated absolute values of the correlations were also recalculated.

### Interobserver and intraobserver variability analyses

We performed ground-truth validation of the manual reference masks by calculating the Dice similarity coefficient (DSC), Hausdorff distance (HD) to assess interobserver and intraobserver variability. For the interobserver analysis, two independent nuclear medicine experts manually resegmented the same 43 lesions used for testing of RECOMIA and nnU-Net model 535 segmentation algorithms (see section Algorithms in Materials and methods), and their masks were compared using DSC and HD. For the intraobserver analysis, newly segmented masks were compared with the same expert’s original masks, also using DSC and HD. Higher DSC suggests greater spatial overlap, whereas lower HD illustrates smaller boundary distance, which suggests closer agreement.

## Results

### nnU-Net demonstrated superior performance compared with RECOMIA for PET-CT solitary lung tumor segmentation

The nnU-Net model 535 and RECOMIA segmentation predictions were compared with the manual masks using radiomics features agreement. Table 2 reports Brown’s method combined p-values based on uncorrected p-values, FDR and Bonferroni-adjusted p-values and the sums of absolute value of correlations. The individual p-values and correlation coefficients used in calculation are provided in S1–S4 Tables. Radiomics-based comparison showed that nnU-Net PET-CT segmentation of solitary lung tumors did not differ significantly from manual segmentations, while RECOMIA showed a significant deviation from the manual one. Therefore, features extracted from nnU-Net predicted masks had closer agreement with manual reference than those extracted from RECOMIA segmentations. These findings indicate the nnU-Net achieved better segmentation accuracy and ability to generate segmentations that more closely resemble expert-delineated tumors. Several representative examples of segmentation outputs from nnU-Net model 535 and RECOMIA segmentation algorithms are shown in Fig 4, S1–S6 Figs.

**Table 2:** Brown’s method combined p-values based on uncorrected p-values, FDR and Bonferroni-adjusted p-values and the sums of absolute value of correlations of nnU-Net model 535 and RECOMIA predicted masks with the manual reference masks.

Segmentation algorithm	Brown's method p- value	Brown's method p-value FDR	Brown's method p-value Bonferroni	Sum of absolute values of correlations
**nnU-Net model 535**	0.481	0.91	1	37.259
**RECOMIA**	< 0.001	< 0.001	< 0.001	16.358

**Fig 4:**
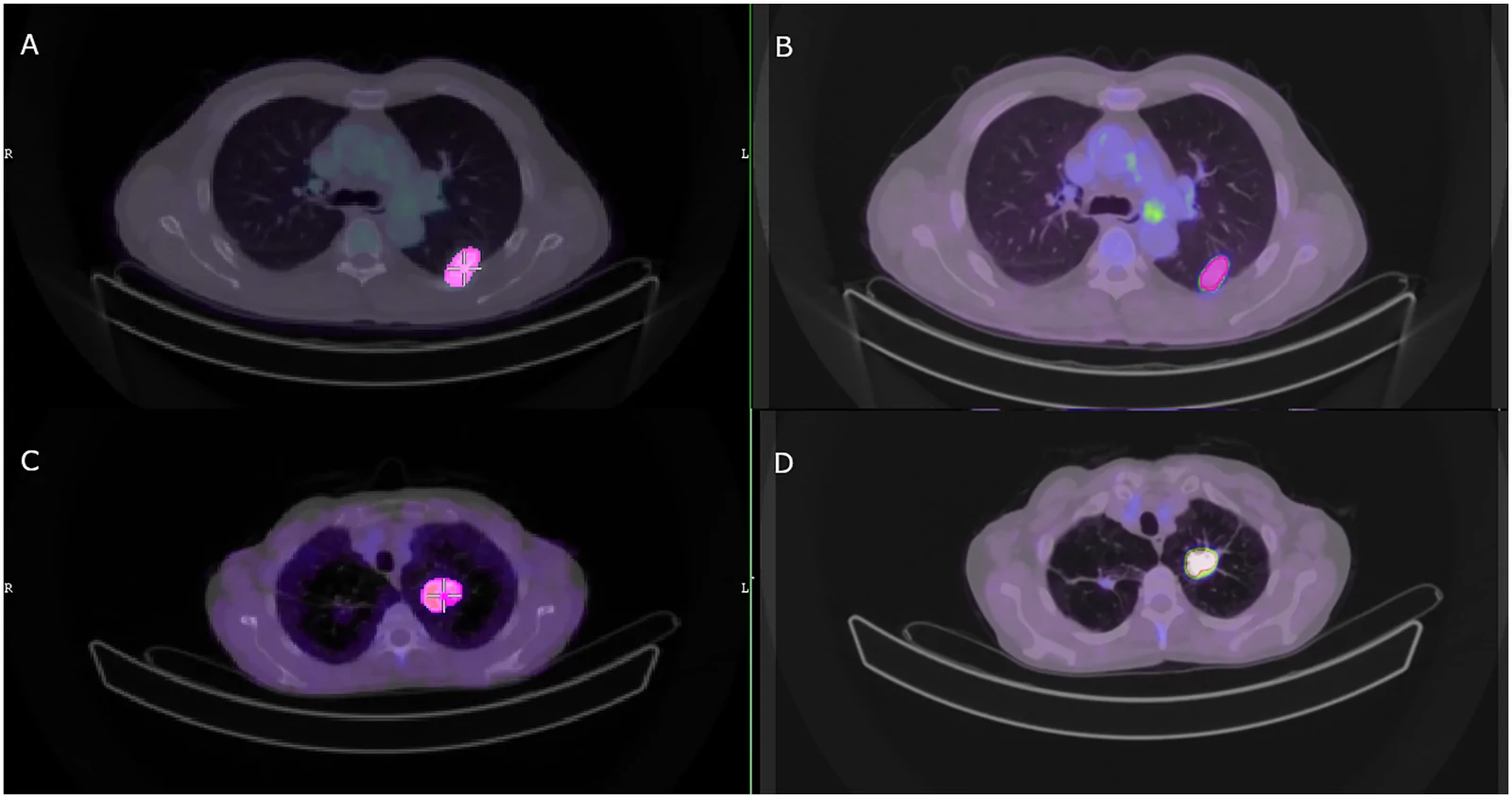
Examples of segmentation outputs from nnU-Net model 535 and RECOMIA segmentation algorithms. Both nnU-Net model 535 (panel A) and RECOMIA (panel B) accurately segmented the lesion in patient p000 with similar shape and volume. For patient p033, only nnU-Net model 535 (panel C) correctly identified the lesion, while RECOMIA (panel D) failed.

### Statistical based accuracy of nnU-Net predictions

To assess the accuracy of our nnU-Net models, we chose four similarity (Jaccard index, Pearson correlation coefficient, mutual information (MI), and normalized mutual information (NMI)) and two dissimilarity (Kullback-Leibler divergence (KLD), L1 norm or Manhattan norm) measures, and then we applied them to the manually and the nnU-Net predicted masks [39] to indicate the extent of (dis)similarity of a pair of individual (mis)matched masks. Because we could not obtain the masks segmented by RECOMIA, we used these metrics only on our nnU-Net datasets. Average (dis)similarity metrics calculated from nnU-Net models 502, 503, 535 and manual masks are shown in Table 3, with details provided in S5–S7 Tables (see Supporting information). We also calculated the Dice Similarity Coefficients (DSC) and Hausdorff Distances (HD) from nnU-Net models 502, 503 and 535 and manual masks (see Table 4 and details in S8 Table), which also display similar results. Altogether, these conventional metric-based results demonstrated that nnU-Net model 535 has the highest quality, as segmentation outputs closely matched the manual reference masks and that model 503 was no better than model 502.

**Table 3:** Average (dis)similarity metrics between nnU-Net models 502, 503, 535 and manual masks.

nnU-Net model	Similarity metrics				Dissimilarity metrics	
	*Jaccard*	*Pearson*	*MI*	*NMI*	*KLD*	*L1 norm*
**535**	0.699	0.820	0.016	1.648	7.124	2377.302
**502**	0.479	0.680	0.351	1.997	3.320	4596
**503**	0.383	0.716	0.353	1.995	1.574	7407

Values are averaged for each model’s respective test set (Model 535 n = 43, Model 502 n = 34, Model 503 n = 22). Not available (NA) values were removed from the calculation. Similarity metrics (higher is better): Jaccard index (Jaccard), Pearson correlation coefficient (Pearson), mutual information (MI), and normalized mutual information (NMI). Dissimilarity metrics (lower is better): Kullback-Leibler divergence (KLD), L1 norm.

**Table 4:** Average Dice Similarity Coefficients (DSC) and Hausdorff Distances (HD) between nnU-Net models 502, 503, 535 and manual masks.

nnU-Net model	DSC	HD
**535**	0.783	2.662
**502**	0.653	30.270
**503**	0.678	4.589

Values are averaged for each model’s respective test set (Model 535 n = 43, Model 502 n = 34, Model 503 n = 22). Failed cases (no mask or double masks) were removed from the calculation. Higher DSC and lower HD indicate better agreement.

### Statistical validation of the proposed radiomics-based comparison method

We further validated the proposed comparison method using ICC and MANOVA. ICC were calculated between nnU-Net model 535, RECOMIA and manual masks, using a two-way mixed-effect model with absolute agreement and mean of k-raters type. We used terminology and the ICC method selection as described in [36]. The results demonstrated moderate agreement between nnU-Net model 535 and manual masks (ICC = 0.658, p < 0.001, 95% CI [0.515, 0.783]) while RECOMIA illustrated poor agreement with manual masks (ICC = 0.448, p < 0.001, 95% CI [0.241, 0.64]).

As the number of segmentation models and cases increased, fewer radiomics features passed the normality test across all radiomics datasets (RECOMIA, nnU-Net model 535 and manual masks), hence MANOVA could not be applied using 41 cases. However, we managed to calculate MANOVA using 28 cases. Two radiomics features passed the normality test across all datasets (RECOMIA, nnU-Net model 535 and manual masks): original_shape_SurfaceVolumeRatio, original_glrlm_RunEntropy. Levene’s test indicated that the assumption of variances homogeneity was violated for original_shape_SurfaceVolumeRatio (F(2, 81) = 2.43, p < 0.001) but not for original_glrlm_RunEntropy (F(2, 81) = 13.707, p = 0.095). Accordingly, we applied Welch’s one-way ANOVA, which revealed a significant difference between the 3 datasets in terms of original_shape_SurfaceVolumeRatio (F(2, 48) = 51.962, p < 0.001) and original_glrlm_RunEntropy (F (2, 48) = 1934.8, p < 0.001). Follow up with pairwise t-test using Benjamini–Hochberg correction showed that for the original_shape_SurfaceVolumeRatio, the RECOMIA’s masks differed significantly from both the manual masks (p < 0.001) and the nnU-Net model 535’s masks (p < 0.001), whereas manual masks and nnU-Net model 535’s masks did not differ (p = 0.85). For original_glrlm_RunEntropy, RECOMIA’s masks also differed significantly from both manual masks (p < 0.001) and nnU-Net model 535’s masks (p < 0.001), while manual and nnU-Net model 535’s masks did not differ significantly (p = 0.96). MANOVA confirmed a significant overall strong effect of segmentation between the 3 datasets: Wilks’ lambda was Λ = 0.029, F (4, 160) = 195.56, p < 0.001, and Pillai’s Trace was V = 0.971, F(4, 162) = 38.25, p < 0.001.

Altogether, ICC, MANOVA and follow up Benjamini–Hochberg-corrected pairwise t-test further confirmed stronger agreement between extracted radiomics features from nnU-Net model 535 predicted masks and manual masks than between RECOMIA predicted masks and manual masks. These results were in accordance with the previous findings, which further supported the higher segmentation accuracy of nnU-Net model 535 and validated the proposed comparison method.

### Robustness analysis

To assess the robustness of the findings, non-parametric bootstrap analysis with replacement was performed with 1,000 iterations. NnU-Net model 535 showed a mean bootstrapped p-value of 0.395. In contrast, RECOMIA demonstrated a markedly smaller mean bootstrapped p-value of < 0.001. Similarly, bootstrap analysis of the sum of absolute values of correlations showed a higher mean value for nnU-Net model 535: 40.559, in comparison with RECOMIA: 19.388. The bootstrap distributions are shown in Fig 5–6. Overall, the ranking of segmentation algorithms remained unchanged following bootstrap analysis, with stronger agreement between extracted radiomics features from nnU-Net model 535 predicted masks and manual masks than between RECOMIA predicted masks and manual masks. These bootstrap findings further confirm that nnU-Net produced more accurate segmentations that better matched expert-delineated tumors.

**Fig 5:**
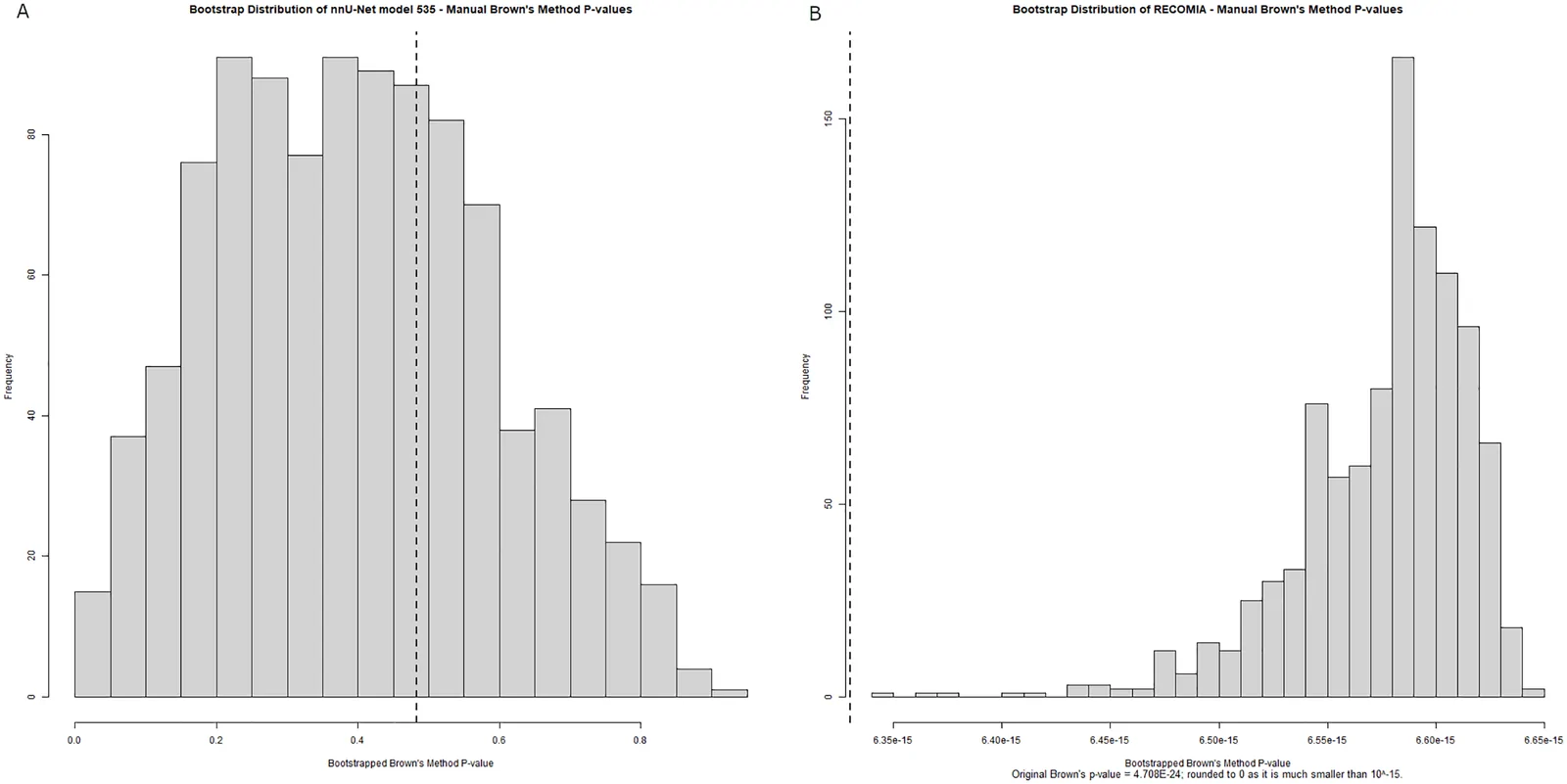
Brown’s method combined p-values bootstrap distribution of nnU-Net model 535 (Panel A) and RECOMIA (Panel B) versus manual segmentation, based on 1,000 non-parametric bootstrap iterations with replacement. The vertical dashed line represents the original Brown’s method p-value. RECOMIA – manual original p-value (4.708 × 10^-24^) was displayed as 0 for visualization.

**Fig 6:**
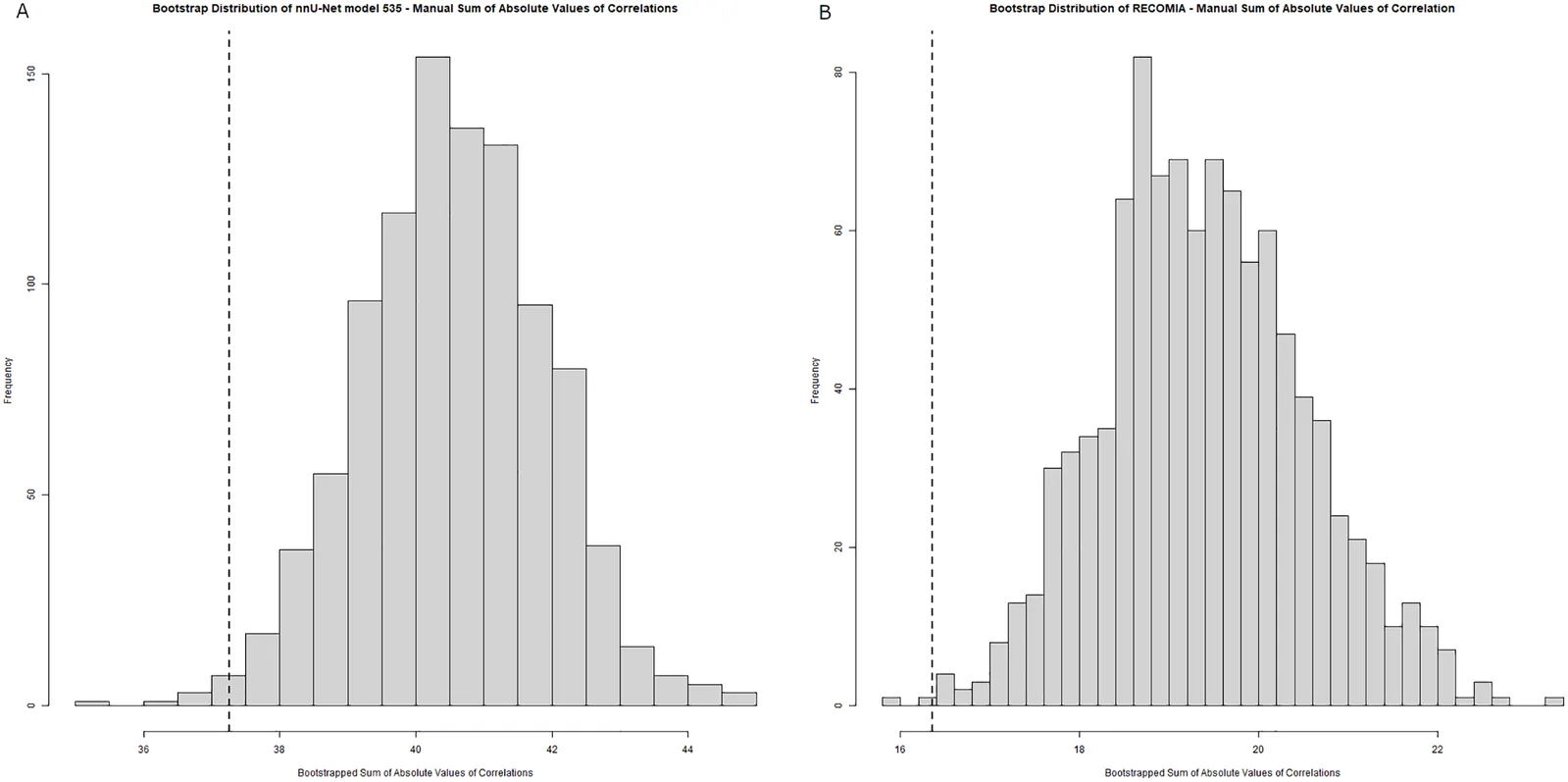
Sum of absolute values of correlation bootstrap distribution of nnU-Net model 535 (Panel A) and RECOMIA (Panel B) versus manual segmentation, based on 1,000 non-parametric bootstrap iterations with replacement. The vertical dashed line represents the original sum of absolute values of correlations.

### Interobserver and intraobserver variability analyses

The reliability of the manual reference masks was evaluated by interobserver and intraobserver variability analyses using the DSC and HD. The mean interobserver agreement between the two experts was 0.917 for DSC and 3.807 for HD. Regarding intraobserver variability, expert 1 shows a mean DSC of 0.852 and HD of 3.765, while expert 2 shows a mean DSC of 0.75 and HD of 4.496. These results indicate a good agreement between observers and repeatability of manual delineation, supporting the use of the manual mask as reference for evaluating the performance of the segmentation algorithms.

## Discussion

In this research, we proposed a radiomics-based comparison method to evaluate segmentation algorithms and applied it to two deep learning-based algorithms nnU-Net and RECOMIA to compare their performance on PET-CT solitary lung tumor segmentation. Radiomics features were extracted from the output masks and compared with those derived from the manually segmented masks as gold standard using combined p-values calculated using Brown’s method and the aggregated absolute values of the correlations. In the independent test cohort, nnU-Net PET-CT segmentation of solitary lung tumors did not differ significantly from manual segmentations, while RECOMIA showed a significant deviation from the manual one. The consistency between radiomics-based results and conventional evaluation metrics, including DSC, HD, similarity and dissimilarity analyses further highlighted the ability of nnU-Net model 535 to generate segmentations that more closely resemble expert-delineated tumors. Furthermore, the DSC value of nnU-Net model 535 was within the range of interobserver and intraobserver variability, while the HD value was even lower in comparison. These results demonstrated that nnU-Net model 535 segmentation performance was comparable to that of human experts. ICC, MANOVA, Benjamini–Hochberg-corrected pairwise t-test and bootstrap analysis further supported the validity and robustness of the findings and proposed comparison method.

Radiomics-based analysis may provide further insight into the impact of tumor characterization on segmentation accuracy. Radiomics features describe tumor morphology and characteristics such as heterogeneity, shape and spatial texture [8–10]. Previous studies demonstrated radiomics features may exhibit varying levels of robustness across feature classes, with shape features generally the best robust, followed by first order features. On the other hand, features quantifying shape, such as Neighboring Gray Tone Difference Matrix (NGTDM) and Gray Level Co-occurrence Matrix (GLCM), usually have worse reproducibility [40]. As radiomics features are sensitive to image acquisition protocol, reconstruction algorithms and extraction software [41], we controlled non-segmentation variability by applying the same testing dataset, radiomics configuration and software to both nnU-Net and RECOMIA.

Comparison methods based on radiomics features not only capture spatial segmentation differences but also variation in clinically relevant information, such as tumor shape, intensity and texture characteristics. Therefore, radiomics agreement may provide complementary information to other conventional metrics such as DSC and HD and may be considered a valuable indicator of segmentation performance [16]. Ultimately, these results suggest that radiomics features, together with our proposed comparison method may serve as valuable complementary tools to conventional metrics for evaluating and comparing segmentation algorithms in clinical and research settings.

The observed difference in performance between two segmentation algorithms could be attributed to several factors. The nnU-Net model was trained directly on our institution’s semi-automated manual masks, created by 7 algorithms: Fuzzy C-means, K-Means, Gaussian mixture model, Active contour, Region growing algorithm, Random walker, Majority voting method of the former ones and graded by experienced nuclear medicine specialists and medical doctors with consistent access to our imaging protocols and relevant clinical information. In comparison, RECOMIA model was trained on a different dataset with annotations from external institutions [18], which may differ in standards, labeling guidelines and interpretation. Furthermore, nnU-Net’s self-configuring nature, which automatically adapts its architecture, preprocessing, and training pipeline [17], enables optimization of the specific characteristics of our data, including scanner types, lesion presentations, and local disease prevalence. In contrast, RECOMIA employs a fixed pre-trained model built on a dataset of 320 images [18]. Without retraining or fine-tuning, it cannot adapt to new data distributions, especially in terms of lesion multiplicity (solitary vs. multi-lesion), lesion size and intensity profiles, which further limits its performance on datasets that differ from its original training domain. Therefore, the observed results may reflect not only the difference in segmentation performance but also differences in domain adaptation factors, such as scanner, protocol compatibility and training data similarity.

The superior performance of nnU-Net can offer numerous benefits for implementation in clinical practice. While higher radiomics agreement does not directly demonstrate improved clinical outcomes, it suggests that a segmentation method could accurately delineate the tumor on PET-CT image, which is essential for deriving reliable quantitative imaging metrics, including total lesion glycolysis (TLG), standardized uptake value (SUV) and metabolic tumor volume (MTV) [42,43]. These parameters may be particularly important to support tumor staging, treatment planning and also to determine patient prognosis and outcome [44,45]. Better segmentation performance may also reduce the time required for manual delineation by nuclear medicine experts in order to calculate the above metrics and improve the reproducibility of measurements across institutions. It is crucial to highlight that in clinical practice, these improvements could permit better radiotherapy planning, surgical decision making and assessment of treatment response, potentially enhance survival in patients with lung cancer [22,46].

Considering real-world applicability, there are meaningful trade-offs between accuracy and efficiency. Importantly, no additional training is required for the end user with the RECOMIA algorithm. The training and execution of RECOMIA were conducted on a Linux desktop equipped with a GeForce RTX 2080 TI graphics card, utilizing a dataset of 320 images. The process took approximately 48 hours [18], while the prediction took on average 1 minute per image. RECOMIA might be a preferable solution in clinical settings without high performance IT infrastructure and where cost-benefits are critical [47]. RECOMIA operates as a cloud-based platform, and computing processes are handled on an external server which eliminates the need for high-performance computers [46] and ensures efficient operation without relying on hospital limited hardware. RECOMIA is also considerably more intuitive and easier to use, especially for senior health professionals who do not want to deal with command line tools, allowing for easier integration into clinical workflows. On the other hand, the nnU-Net algorithm requires additional imaging resources and costs for local training and computing. The nnU-Net model 535 was run on a local High Performance Computing (HPC) cluster (8 vcpus, 4 GiB memory, NVIDIA Tesla A100 GPU (80GB VRAM)). The training time was approximately 9 hours, the average prediction time was approximately 3 seconds for each image, both are much faster than RECOMIA because of the higher performance setup. In contrast, nnU-Net models 502 and 503 were run on a Standard NC6s v3 virtual machine (6 vcpus, 112 GiB memory, NVIDIA Tesla V100 GPU (16 GB VRAM)) in Microsoft Azure. Despite the utilization of a substantially higher-performance GPU relative to GeForce RTX 2080 TI, the training for both the nnU-Net models 502 and 503 took approximately 48 hours (similar to RECOMIA) and their average prediction time was approximately 4 minutes per image, approximately four times slower.

Emergent developments in nnU-Net frameworks offer promising solutions to several limitations identified in this study. Many recent lightweight pretrained nnU-Net models such as TotalSegmentator and MOOSE, as well as streamlined 3D Slicer module integration with simplified deployment, now allow segmentation and radiomics feature extraction to be also performed also by end users without extensive technical expertise [48,49]. These advances narrow the accessibility gap between RECOMIA and locally deployed nnU-Net segmentation algorithms, making the latter increasingly accessible in both research and clinical settings. Furthermore, continual-learning algorithms, for example Lifelong nnU-Net may allow generalizability across scanners, protocols and patient populations, directly addressing the domain shift effects observed here [50]. This can help to enhance robustness of segmentation models in real world clinical environments.

This study has several limitations that must be acknowledged. Firstly, we performed a plain methodological investigation, without any direct clinical correlation with lung cancer-related outcomes, such as diagnosis, treatment response and survival. Therefore, although our proposed radiomics-based comparison method demonstrated differences in performance between segmentation algorithms, the clinical relevance of this remains to be verified. Secondly, the datasets used in this study may not fully represent the wide variability seen in clinical practice, especially in terms of the scanner spectrum, acquisition protocols and lesion characteristics. Radiomics features are also known to exhibit redundancy, collinearity and varying levels of robustness [40,51], which may influence the statistical significance of the observed differences. Although Brown's method was applied to account for feature correlation when combining p-values, residual dependency of the radiomics features may still affect the results. Furthermore, the removal of non-normally distributed features may introduce bias and alter feature representation.

A further limitation of this study is that Wavelet and Laplacian-of-Gaussian features were not evaluated as RECOMIA platform does not provide the segmentation masks required for extracting these filtered image features. To ensure a consistent comparison across all segmentation methods, we restricted the comparison to the original set radiomics features that are available for all methods. We also did not conduct external prospective validation or detailed subgroup analyses (e.g., by scanner brands, lesion size), which could reveal different context-specific performance patterns.

We advise future studies to evaluate both methods under matched training and testing conditions to better isolate the domain adaptation effects. We also recommend continuing research with large scale, multi-center PET-CT datasets, which may provide more generalizable results. Further research should apply this comparison method to other algorithms (such as semisupervised conditional nnU-Net [52] and efficient U-Net [53]) to assist the selection for specific clinical and research needs. We suggest the use of a transformation method to convert non-Gaussian data to Gaussian-like shapes (for example Box Cox transformation) or other calculation methods that do not assume the normality of the data (for example Empirical Brown’s method) to generate a more reliable p-value. Further studies having access to the predicted masks from all segmentation methods should investigate whether the inclusion of filtered-image features (such as Wavelet and Laplacian-of-Gaussian) could provide additional value for performance evaluation. Further nnU-Net research should be performed to continue optimizing the architecture for better computational efficiency [54,55] and develop more accessible user-interfaces. We also recommend further RECOMIA research to optimize the accuracy.

## Conclusions

In this research, we proposed a radiomics-based method to evaluate segmentation algorithms and applied it to compare the performance of two deep learning-based algorithms nnU-Net and RECOMIA for PET-CT solitary lung tumor segmentation. This approach ranks algorithms according to the agreement between radiomics features extracted from automated segmentation outputs and those derived from manual reference masks. The proposed method may serve as valuable complementary tools to conventional metrics such as DSC and HD for evaluating and comparing segmentation algorithms. nnU-Net demonstrated significantly closer agreement with manual reference masks than the RECOMIA model, as measured by radiomics-based analysis and validated by conventional metrics. The superior performance of nnU-Net highlights the importance of training data alignment and model adaptability in achieving accurate and reliable segmentation in medical imaging. However, the training requirements and additional computational demands of nnU-Net may limit its applicability in some clinical environments. Despite lower accuracy, RECOMIA remains valuable because of its efficiency and intuitiveness. These findings provide valuable guidance for selecting and deploying segmentation algorithms in real-world clinical and research settings. Further research is necessary to validate the proposed method with large scale, multi-center datasets and across a wide variety of segmentation algorithms.

## Supporting information

S1 Table Feature-wise Wilcoxon p-values and correlations between RECOMIA, nnU-Net model 535 and manual mask’s radiomics features. P-values are shown before FDR and Bonferroni correction. Not available (NA) values were removed from the calculation of sums of absolute value of correlations.
(DOCX)

S2 Table Feature-wise p-values used in calculation of nnU-Net model 535 Brown’s method combined p-value. For each radiomics parameter, the table reports Wilcoxon signed rank test p-values calculated using segmentation algorithm’s predicted masks and manual masks.
(DOCX)

S3 Table Feature-wise p-values used in calculation of RECOMIA Brown’s method combined p-value. For each radiomics parameter, the table reports Wilcoxon signed rank test p-values calculated using segmentation algorithm’s predicted masks and manual masks.
(DOCX)

S4 Table Feature-wise correlations used in calculation of nnU-Net model 535 and RECOMIA sum of absolute values of correlation. For each radiomics parameter, the table reports correlation (cor) between segmentation algorithm’s predicted masks and manual masks.
(DOCX)

S5 Table Per-case (dis)similarity metrics between from nnU-Net model 535 and manual masks. Similarity metrics (higher is better): Jaccard index (Jaccard), Pearson correlation coefficient (Pearson), mutual information (MI), and normalized mutual information (NMI). Dissimilarity metrics (lower is better): Kullback-Leibler divergence (KLD), L1 norm.
(DOCX)

S6 Table Per-case (dis)similarity metrics calculated from nnU-Net model 502 and manual masks. Similarity metrics (higher is better): Jaccard index (Jaccard), Pearson correlation coefficient (Pearson), mutual information (MI), and normalized mutual information (NMI). Dissimilarity metrics (lower is better): Kullback-Leibler divergence (KLD), L1 norm. Not available (N/A) values were removed from the calculation of average.
(DOCX)

S7 Table Per-case (dis)similarity metrics calculated from nnU-Net model 503 and manual masks. Similarity metrics (higher is better): Jaccard index (Jaccard), Pearson correlation coefficient (Pearson), mutual information (MI), and normalized mutual information (NMI). Dissimilarity metrics (lower is better): Kullback-Leibler divergence (KLD), L1 norm. Not available (N/A) values were removed from the calculation of average.
(DOCX)

S8 Table Dice Similarity Coefficients (DSC) and Hausdorff Distances (HD) between nnU-Net models 502, 503, 535 and manual masks. Failed cases (no mask or double masks) were removed from the calculation. NM = no mask, DM = double masks and TR: used for training.
(DOCX)

S1 Fig Examples of segmentation outputs from nnU-Net model 535 (left) and RECOMIA (right) segmentation algorithms. Both masks demonstrate the correct lesion with similar shape and volume (p017).
(TIF)

S2 Fig Examples of segmentation outputs from nnU-Net model 535 (left) and RECOMIA (right) segmentation algorithms. Both masks demonstrate the correct lesion with similar shape and volume (p020).
(TIF)

S3 Fig Examples of segmentation outputs from nnU-Net model 535 (left) and RECOMIA (right) segmentation algorithms. Both masks demonstrate the correct lesion but neither covers the whole tumor (p001).
(TIF)

S4 Fig Examples of segmentation outputs from nnU-Net model 535 (left) and RECOMIA (right) segmentation algorithms. Both masks demonstrate the correct lesion but neither covers the whole tumor (p021).
(TIF)

S5 Fig Examples of segmentation outputs from nnU-Net model 535 (left) and RECOMIA (right) segmentation algorithms. Both masks demonstrate the correct lesion. Only NnU-Net model 535’s mask covers the whole tumor. (p015).
(TIF)

S6 Fig Examples of segmentation outputs from nnU-Net model 535 (left) and RECOMIA (right) segmentation algorithms. Both masks demonstrate the correct lesion. Only NnU-Net model 535’s mask covers the whole tumor. (p037).
(TIF)
