# Supplementary material for: Assessment of algorithmic performance in PET-CT segmentation of solitary lung tumors
Source: PLoS One. 2026 Nov 5;21(11):e0360849. doi: 10.1371/journal.pone.0360849 (PMC13739142; doi:10.1371/journal.pone.0360849)
Supplement: Supplementary file 1 [file pone.0360849.s016.pdf]

Assessment of Algorithmic Performance in PET-CT Segmentation of Solitary Lung Tumors

Response to Reviewers

Academic Editor

Journal Requirements:

When submitting your revision, we need you to address these additional requirements.

E.1. Please ensure that your manuscript meets PLOS ONE's style requirements, including those for file naming. The PLOS ONE style templates can be found at

https://journals.plos.org/plosone/s/file?id=wjVg/PLOSOne_formatting_sample_main_body.pdf
and https://journals.plos.org/plosone/s/file?id=ba62/PLOSOne_formatting_sample_title_authors_affiliations.pdf

- **Agree – revised:** Thank you for this important suggestion. We have revised the manuscript accordingly.

E.2. The ethical approval number(s) listed in the manuscript and/or submission metadata does not match the approval number on the ethical approval document you provided. Please ensure that all approval numbers are correct.

- **Agree – revised:** Thank you for this important comment. This is the correct Protocol ID (DE RKEB/IKEB-nél) 6504-2023, 13th September 2023). We have revised the manuscript accordingly.

E.3. Please note that PLOS One has specific guidelines on code sharing for submissions in which author-generated code underpins the findings in the manuscript. In these cases, we expect all author-generated code to be made available without restrictions upon publication of the work. Please review our guidelines at https://journals.plos.org/plosone/s/materials-and-software-sharing#loc-sharing-code and ensure that your code is shared in a way that follows best practice and facilitates reproducibility and reuse.

- **Agree – revised:** Thank you for this important comment. The codes are available at https://github.com/opgabor/nnUNet-vs-RECOMIA. We have requested for correction in the cover letter and sent you an Updated Data statement via email.
- Updated Data statement: “The data analyzed in this study, including sensitive patient information, will be made available upon reasonable request from the University of Debrecen Data Protection Officer in accordance with applicable ethical and data

protection regulations. Address: University of Debrecen Data Protection Officer, Kassai út campus, H-4028 Debrecen, Kassai u. 26. Chancellery IV, ground floor 34, website: https://unideb.hu/en/data-protection-officer-dpo, email: adatvedelmi.tisztviselo@unideb.hu, phone: +36 52,512,700/74465. The source codes are available at https://github.com/opgabor/nnUNet-vs-RECOMIA."

E.4. Thank you for stating the following in the Acknowledgments Section of your manuscript:

"Project no. TKP2021-NKTA-34 has been implemented with the support provided by the Ministry of Culture and Innovation of Hungary from the National Research, Development, and Innovation Fund, financed under the TKP2021-NKTA funding scheme."

We note that you have provided funding information that is not currently declared in your Funding Statement. However, funding information should not appear in the Acknowledgments section or other areas of your manuscript. We will only publish funding information present in the Funding Statement section of the online submission form.

Please remove any funding-related text from the manuscript and let us know how you would like to update your Funding Statement. Currently, your Funding Statement reads as follows:

"Project no. TKP2021-NKTA-34 has been implemented with the support provided by the Ministry of Culture and Innovation of Hungary from the National Research, Development, and Innovation Fund, financed under the TKP2021-NKTA funding scheme."

Please include your amended statements within your cover letter; we will change the online submission form on your behalf.

- **Agree – revised:** Thank you for this important comment. We have deleted the funding from the Acknowledgments section. We have included the updated Funding statement in our cover letter.

E.5. Thank you for stating the following financial disclosure:

"Project no. TKP2021-NKTA-34 has been implemented with the support provided by the Ministry of Culture and Innovation of Hungary from the National Research, Development, and Innovation Fund, financed under the TKP2021-NKTA funding scheme."

Please state what role the funders took in the study. If the funders had no role, please state: "The funders had no role in study design, data collection and analysis, decision to publish, or preparation of the manuscript."

If this statement is not correct you must amend it as needed.

Please include this amended Role of Funder statement in your cover letter; we will change the online submission form on your behalf.

- **Agree – revised:** Thank you for this important comment. The mentioned sentence belongs to the Funding statement, not the financial disclosure. I am sorry for this misunderstanding. The request for correction has been included in the cover letter.
- Updated Financial Disclosure Statement "Project no. TKP2021-NKTA-34 has been implemented with the support provided by the Ministry of Culture and Innovation of Hungary from the National Research, Development, and Innovation Fund, financed under the TKP2021-NKTA funding scheme. The funders had no role in study design, data collection and analysis, decision to publish, or preparation of the manuscript. Scanomed Ltd., provided support in the form of salaries for authors Márton Mikó, Bence Farkas, but did not have any additional role in the study design, data collection and analysis, decision to publish, or preparation of the manuscript. The specific roles of these authors are articulated in the 'author contributions' section."

E.6. In the online submission form you indicate that your data is not available for proprietary reasons and have provided a contact point for accessing this data. Please note that your current contact point is a co-author on this manuscript. According to our Data Policy, the contact point must not be an author on the manuscript and must be an institutional contact, ideally not an individual. Please revise your data statement to a non-author institutional point of contact, such as a data access or ethics committee, and send this to us via return email. Please also include contact information for the third party organization, and please include the full citation of where the data can be found.

- **Agree – revised:** Thank you for this important comment. The request for correction has been included in the cover letter and the updated Data statement has been sent to you through return email.
- Updated Data statement: "The data analyzed in this study, including sensitive patient information, will be made available upon reasonable request from the University of Debrecen Data Protection Officer in accordance with applicable ethical and data protection regulations. Address: University of Debrecen Data Protection Officer, Kassai út campus, H-4028 Debrecen, Kassai u. 26. Chancellery IV, ground floor 34, website: https://unideb.hu/en/data-protection-officer-dpo, email: adatvedelmi.tisztviselo@unideb.hu, phone: +36 52,512,700/74465. The source codes are available at https://github.com/opgabor/nnUNet-vs-RECOMIA."

E.7. Thank you for stating the following in the Competing Interests section:

The authors declare that they have no competing interests.

We note that one or more of the authors are employed by a commercial company: “Scanomed Ltd., Debrecen, Hungary”

1. Please provide an amended Funding Statement declaring this commercial affiliation, as well as a statement regarding the Role of Funders in your study. If the funding organization did not play a role in the study design, data collection and analysis, decision to publish, or preparation of the manuscript and only provided financial support in the form of authors' salaries and/or research materials, please review your statements relating to the author contributions, and ensure you have specifically and accurately indicated the role(s) that these authors had in your study. You can update author roles in the Author Contributions section of the online submission form.

Please also include the following statement within your amended Funding Statement.

“Scanomed Ltd., provided support in the form of salaries for authors [insert relevant initials], but did not have any additional role in the study design, data collection and analysis, decision to publish, or preparation of the manuscript. The specific roles of these authors are articulated in the ‘author contributions’ section.”

If your commercial affiliation did play a role in your study, please state and explain this role within your updated Funding Statement.

2. Please also provide an updated Competing Interests Statement declaring this commercial affiliation along with any other relevant declarations relating to employment, consultancy, patents, products in development, or marketed products, etc.

Within your Competing Interests Statement, please confirm that this commercial affiliation does not alter your adherence to all PLOS ONE policies on sharing data and materials by including the following statement: "This does not alter our adherence to PLOS ONE policies on sharing data and materials.” (as detailed online in our guide for authors http://journals.plos.org/plosone/s/competing-interests) . If this adherence statement is not accurate and there are restrictions on sharing of data and/or materials, please state these. Please note that we cannot proceed with consideration of your article until this information has been declared.

Please include both an updated Funding Statement and Competing Interests Statement in your cover letter. We will change the online submission form on your behalf.

- **Agree – revised:** Thank you for this important comment. We have stated the request for corrections and updated the Funding Statement and Competing Interests Statement in the cover letter.

- Updated Funding statement: “Project no. TKP2021-NKTA-34 has been implemented with the support provided by the Ministry of Culture and Innovation of Hungary from the National Research, Development, and Innovation Fund, financed under the TKP2021-NKTA funding scheme. The funders had no role in study design, data collection and analysis, decision to publish, or preparation of the manuscript. Scanomed Ltd., provided support in the form of salaries for authors Márton Mikó, Bence Farkas, but did not have any additional role in the study design, data collection and analysis, decision to publish, or preparation of the manuscript. The specific roles of these authors are articulated in the ‘author contributions’ section.”
- Updated Competing interest statement: “Scanomed Ltd., provided support in the form of salaries for authors Márton Mikó, Bence Farkas, but did not have any additional role in the study design, data collection and analysis, decision to publish, or preparation of the manuscript. Scanomed Ltd., affiliation does not alter our adherence to PLOS ONE policies on sharing data and materials.”

E.8. Your ethics statement should only appear in the Methods section of your manuscript. If your ethics statement is written in any section besides the Methods, please delete it from any other section.

- **Agree – revised:** Thank you for this important comment. Ethics statement has been deleted from the Declarations and added to “Study design and Ethical approval” point in Material and Methods.

E.9. We notice that your supplementary tables are included in the manuscript file. Please remove them and upload them with the file type 'Supporting Information'. Please ensure that each Supporting Information file has a legend listed in the manuscript after the references list.

- **Agree – revised:** Thank you for this important suggestion. We have removed the supplementary tables and added them to the Supporting information file. Each table in Supporting Information file has a legend listed in the manuscript after the references list.

E.10. If the reviewer comments include a recommendation to cite specific previously published works, please review and evaluate these publications to determine whether they are relevant and should be cited. There is no requirement to cite these works unless the editor has indicated otherwise.

- **Agree:** Thank you for this important comment.

Additional Editor Comments:

Dear Authors, please revise the paper according to the suggestions provided by the Reviewers, particularly explaining the clinical implications and usefulness of your results, and clarifying the real aim of the paper (to assess radiomic reproducibility or to find the most accurate segmentation software?). Moreover, provide more detailed discussion on radiomic parameters.

- **Agree – revised:** Thank you for this important comment. We have revised the manuscript according to the reviewers' suggestions. We have added explanations about clinical implications, usefulness of the results, clarified the real aim of the paper and provided detailed discussion on radiomics parameters. We have added the OCRID of coauthors as required.

Best regards
Carmelo Caldarella

Reviewer #1: This is a paper evaluating different segmentation methods. From my point of view, it is not clear whether the aim is to assess radiomic reproducibility or to find the most accurate segmentation software. Here are my further comments:

R1.1. "accurate diagnosis requires careful examination of PET-CT images by a skilled nuclear medicine expert, which is not only tedious but also extremely time-consuming". I do not agree, since personally I need 10-15 min for lung cancer PET reporting assessment. The above-mentioned period can be true when dealing with contrast-enhanced PET/CT or tumor segmentation or response assessment based on standardized criteria.

- **Agree – revised:** Thank you for your important suggestion. We agree that the original statement may not accurately reflect routine clinical PET-CT interpretation. We have revised this statement in the Introduction to according to your suggestion.

R1.2. "radiomic features offer a comprehensive characterization of the entire tumor, which is usually heterogeneous". I would rather say that "radiomic CAN offer"

- **Agree – revised:** Thank you for this important suggestion. We have revised the manuscript accordingly.

R1.3. "Currently, there is a scarcity of published literature using radiomics features to compare the performance of different tumor segmentation algorithms on PET-CT images". I hope that this is wrong since it seems that you segment tumor based on radiomic that is completely not clinically acceptable due to missing, strongly validated parameters. I think that the authors wanted to assess whether different segmentations system can obtain the same rame radiomic values.

- **Agree – revised:** Thank you for this important comment. It is correct that we want to assess whether different segmentations system can obtain similar radiomic values to manual reference masks. We would like to clarify that our study did not perform segmentation based on radiomics features. We have revised that sentence and added clarifications to the Introduction section to avoid confusion, according to your suggestion.

R1.4. In the whole paper, use proper radiopharmaceutical nomenclature according to EANM

- **Agree – revised:** Thank you for this important comment. We have revised the manuscript according to your suggestion.

R1.5. Reduce the high number of figures by moving some to supplements.

- **Agree – revised:** Thank you for your important suggestion. We have moved 6 figures to Supplements section.

R1.6. From the results section, it is not clear which one was the more precise segmentation compared to the manual one, as in my eyes (DSC), it is a more important parameter than the radiomic one.

- **Partially Agree – revised:** Thank you for this important comment. We agree that the Results section should clearly indicate which segmentation was more precise, in comparison with the manual one. We have revised the Results section to highlight the superior performance of nnU-Net.
- The novel objective of this study was to develop a complementary comparison method, based on radiomics features extracted from the segmentation outputs, which take into account the tumor shape, intensity, and texture characteristics, in addition to classical metrics when comparing segmentation algorithms. We want to highlight that Akramova and Watanabe demonstrated that radiomics is better evaluation tool than DSC for tumor segmentation performance, as it also incorporates information of tumor shape, intensity, and texture characteristics.
- Please see reference below:
    - Akramova, R., & Watanabe, Y. (2024). Radiomics as a measure superior to common similarity metrics for tumor segmentation performance evaluation. *Journal of applied clinical medical physics*, *25*(8), e14442. https://doi.org/10.1002/acm2.14442

R1.7. The results section is more similar to a table index, the authors should start presenting the main results of the whole paper.

- **Agree – revised:** Thank you for this important suggestion. We have revised and added the main results to the Results section.

R1.8. "This study demonstrated that nnU-Net significantly outperforms RECOMIA in terms of PET-CT solitary lung tumor segmentation, as evidenced by closer agreement of radiomic features with manual reference masks". In my eyes, the fact that radiomic is more reproducible il much more less important than the segmentation volume DSC. This should be consider by the authors who should modify the paper structure/results presentation accordingly. Otherwise, the study would focus only on radiomic features reproducibility among different segmentation software, which, however, cannot be considered to assess a segmentation software.

- **Partially Agree – revised:** Thank you for this important comment. We appreciate the suggestion and we agree that conventional metrics such as DSC and HD remain essential for comparing the performance of segmentation algorithms. We would like to clarify that our goal was not to achieve reproducibility of radiomic parameters.

Rather, the novel objective of this study was to develop a complementary comparison method, based on radiomics features extracted from the segmentation outputs, which take into account the tumor shape, intensity, and texture characteristics, in addition to classical metrics when comparing segmentation algorithms.

- Radiomic features agreement-based evaluation had already been used in literature for segmentation comparison. Previously, Philip et al. have applied statistical analyses (ICC, CP, TDI, CIA, LoA) on 92 radiomic features to compare performance manual and six semi-automatic segmentation, based on radiomic features agreement with manual mask. Therefore, in our view, in addition to conventional metrics such as DSC and HD, radiomics could also be used to assess performance of segmentation algorithms. We have revised the manuscript accordingly.
- Please see reference below:
    - Philip, M. M., Watts, J., Moeini, S. N. M., Musheb, M., McKiddie, F., Welch, A., & Nath, M. (2024). Comparison of semi-automatic and manual segmentation methods for tumor delineation on head and neck squamous cell carcinoma (HNSCC) positron emission tomography (PET) images. *Physics in medicine and biology*, *69*(9), 10.1088/1361-6560/ad37ea. https://doi.org/10.1088/1361-6560/ad37ea

Reviewer #2: This is a well-designed and executed study to compare two recently developed AI-based autosegmentation tools. The analysis is very comprehensive, and the results are consistent with the conclusions. I have only a few comments as shown below.

General comments

R2.1. A table summarizing the number of cases used for training and testing of two algorithms will help readers to understand the method more easily.

- **Agree – revised:** Thank you for this important suggestion. We have added **Table 1** demonstrating the PET-CT images allocations for training and testing, together with model output.

R2.2. Radiomics features are used for performance comparison. However, the discussion of the results on radiomics is missing.

- **Agree – revised:** Thank you for this important comment. We have added discussion of the results on radiomics according to your suggestion.

R2.3. Furthermore, the introduction implies that radiomics application is main aim of this study by evidenced by one paragraph devoted to this topic. The first sentence of the last paragraph in Introduction gives the readers a false impression about the primary goal of this paper.

- **Agree – revised:** Thank you for this important comment. We have reworded that sentence to avoid misunderstanding.

R2.4. Please add more details about the radiomics feature calculations. What kind of filters were applied? Some publications show that the wavelet-based radiomics features are more sensitive to segmentation differences. See, for example, the following reference: Akramova, R. and Y. Watanabe, Radiomics as a measure superior to common similarity metrics for tumor segmentation performance evaluation. J Appl Clin Med Phys, 2024. 25(8): p. e14442.

- **Agree – revised:** Thank you for this important suggestion. We have added details of radiomics features calculations accordingly.

Reviewer #3: In this paper, the authors compared the performance of two deep learning-based algorithms (mnU-Net and RECOMIA) in the segmentation of solitary lung tumor at PET images after the generation of radiomic features, these latter able to better characterize lung tumor biology.

After comparative tests, the authors conclude that radiomic features generated by mnU-Net are more similar to those derived by manual segmentation, suggesting a superiority of this segmentation algorithm.

R3.1. This manuscript is a pure methodological paper and no clinical correlation with radiomics data and features of lung cancer are provided. Which is the clinical impact of a better segmentation? Which parameters may be generated by an optimal segmentation? How it may be useful in diagnosis and monitoring of lung cancer?

- **Agree – revised:** Thank you for this important comment. We have added details of clinical correlation, parameters may be beneficially impacted by optimal segmentation, usefulness in diagnosis, radiotherapy planning and assessment of treatment response in the Discussion section. We have revised the limitations of this study according to your suggestion.

R3.2. Was the study retrospective or prospective? Please clarify

- **Agree – revised:** Thank you for this important suggestion. We have added details of study design to "Study design and Ethical approval" point in Material and Methods section.

R3.3. The projections reported in Figures 4-11 for two methods of analysis are often different. Please choose the three spatial projections for the two methods.

- **Agree – revised:** Thank you for this important suggestion. Because three identical spatial projections could not be obtained from RECOMIA system, to ensure harmonization, we have revised the figures showing one representative spatial projection for both methods.
