# Supplementary material for: Assessment of algorithmic performance in PET-CT segmentation of solitary lung tumors
Source: PLoS One. 2026 Nov 5;21(11):e0360849. doi: 10.1371/journal.pone.0360849 (PMC13739142; doi:10.1371/journal.pone.0360849)
Supplement: Supplementary file 2 [file pone.0360849.s017.pdf]

Reviewer #4:

Major Comments

R4.1. The comparison between nnU-Net and RECOMIA is not methodologically balanced

The most important concern is that the two algorithms were evaluated under substantially different conditions.

The nnU-Net model was trained and optimized using local institutional data, scanner characteristics, acquisition protocols, and expert-approved masks derived from the same institution. In contrast, RECOMIA was evaluated as an externally pretrained model without fine-tuning on the local dataset.

Therefore, the study may primarily reflect:

domain adaptation differences,

scanner/protocol compatibility,

training data similarity,

rather than intrinsic superiority of one segmentation algorithm over another.

This issue should be discussed much more explicitly in the manuscript, and the conclusions should be substantially toned down.

- **Agree – revised:** Thank you for your important suggestions. We have revised the Discussion section to acknowledge that the observed results may reflect domain adaptation, scanner and protocol compatibility and training data similarity. We have also toned down the conclusions accordingly.

R4.2. The central assumption that "radiomics agreement reflects segmentation quality" remains insufficiently validated

The manuscript proposes radiomics feature agreement as a surrogate marker for segmentation quality. While this is an interesting concept, the biological and methodological validity of this assumption remains uncertain.

Radiomics features are highly sensitive to:

voxel spacing,

reconstruction algorithms,

smoothing,

discretization methods,

noise,

partial volume effects.

Therefore, differences in radiomics features do not necessarily indicate poorer segmentation quality.

The authors should better justify:

why radiomics agreement should be considered a reliable indicator of segmentation performance,

which radiomics features are expected to be robust,

and how non-segmentation-related variability was controlled.

Currently, the manuscript risks conflating:

radiomics reproducibility,

with

segmentation accuracy.

- **Partially agree – revised:** Thank you for this important comment. We have revised the Discussion to better explain the proposed radiomics agreement-based evaluation method.
- Akramova and Watanabe demonstrated that radiomics is better evaluation tool than DSC for tumor segmentation performance. Radiomics features exhibited greater sensitivity to segmentation changes than DSC, and it also incorporates information of tumor shape, intensity, and texture characteristics.
- Regarding feature robustness, Haaburger et al demonstrated radiomics feature robustness may vary across feature classes, with shape features generally the best robust, followed by first order features. On the other hand, features quantifying shape, such as ngtdm and glcm, usually have worse reproducibility. These findings have been added to the Discussion.
- Finally, we have minimized the non-segmentation-related variability by using the identical dataset (details see Datasets section) and radiomics configuration settings to evaluate the two segmentation algorithms. Therefore, the differences in radiomics features likely indicate segmentation output quality, rather than variations in image acquisition or feature extraction protocol.

- Akramova, R. and Y. Watanabe, *Radiomics as a measure superior to common similarity metrics for tumor segmentation performance evaluation*. J Appl Clin Med Phys, 2024. **25**(8): p. e14442 DOI: 10.1002/acm2.14442.
- Haarburger, C., et al., *Radiomics feature reproducibility under inter-rater variability in segmentations of CT images*. Sci Rep, 2020. **10**(1): p. 12688 DOI: 10.1038/s41598-020-69534-6.

R4.3. Lack of robust ground-truth validation

Manual segmentation was treated as the gold standard; however:

interobserver variability was not evaluated,

intraobserver reproducibility was not reported,

consensus procedures were insufficiently described.

This is particularly important in PET tumor delineation, where contour variability can be substantial because of:

respiratory motion,

inflammatory uptake,

partial volume effects,

threshold ambiguity.

Without demonstrating agreement between human experts, the reliability of the reference masks remains uncertain.

The manuscript would be strengthened by reporting:

manual-manual DSC,

ICC between observers,

or consensus methodology.

- **Agree – revised:** Thank you for your important suggestions. We have added the interobserver and intraobserver variability analyses accordingly.

R4.4. Radiomics methodology requires additional detail and justification

The radiomics analysis appears relatively simplified:

no wavelet filters,

no Laplacian-of-Gaussian features,

no harmonization strategy,

no robustness analysis,

no IBSI compliance discussion.

Furthermore, 107 radiomics features were analyzed without sufficient discussion of:

feature redundancy,

collinearity,

feature stability.

The statistical framework therefore may overestimate the significance of observed differences.

- **Partially agree – revised:** Thank you for your important suggestions.
- Wavelet and Laplacian-of-Gaussian features were not included because RECOMIA only provides us with the extracted radiomics features but not the corresponding segmentation masks required to generate filtered radiomics features. To ensure a consistent comparison across all segmentation methods, the analysis was therefore restricted to the original set radiomics features that are available for all methods. We have acknowledged this in the limitation paragraph of the Discussion section.
- Harmonization strategy was not applied because all PET/CT examinations were acquired using the same scanner and protocol (details see Datasets section). Harmonization strategies are intended to reduce biases from scanner and protocol variabilities according to Mali et al. Additionally, we used the identical dataset and radiomics configuration settings to evaluate the two segmentation algorithms.
- We have added bootstrap-based robustness analysis. The ranking of segmentation algorithms remained unchanged following bootstrap analysis, which further supports the robustness of the findings.
- We have added information regarding IBSI compliance feature extraction to the Radiomics section.
- We have added discussion regarding radiomics feature redundancy, collinearity and feature stability to the Discussion section.

- Mali, S.A., et al., *Making Radiomics More Reproducible across Scanner and Imaging Protocol Variations: A Review of Harmonization Methods.* J Pers Med, 2021. **11**(9) DOI: 10.3390/jpm11090842.

R4.5. Statistical concerns

Several aspects of the statistical analysis require clarification:

a) Multiple testing and feature dependency

Radiomics features are highly correlated. Although Brown's method was used, it remains unclear whether this sufficiently accounts for feature dependency.

b) Feature exclusion based on normality

The authors removed non-normally distributed features. However, radiomics features are commonly non-Gaussian. Excluding these variables may introduce bias and alter feature representation.

Please clarify:

how many features were excluded,

which feature classes were affected,

and whether sensitivity analyses were performed.

c) Interpretation of aggregate metrics

The biological and clinical interpretation of:

summed absolute correlations,

combined p-values,

remains somewhat unclear.

- **Agree – revised:** Thank you for your important suggestions.
- a) We agree that radiomics features are highly correlated. To address this issue, we used Brown's method specifically because it extends Fisher's method, allow us to combine dependent p-values (Brown). However, the residual dependency of radiomics features may still affect the results. The sufficiency of this method to account for feature dependency requires further statistical analysis, which is outside of the scope of this study. We have acknowledged this in the limitation paragraph of the Discussion section.

- b) We agree that removing non-normally distributed features may introduce bias and alter feature representation. However, this is necessary based on Brown's method multivariate normality assumption. We have acknowledged this in the limitation paragraph of the Discussion section and suggest further research using transformation method to convert non-Gaussian data to Gaussian-like shapes (for example Box Cox transformation) or other calculation methods that do not assume the normality of the data (for example Empirical Brown's method) to generate a more reliable p-value. Details regarding excluded features are available in Table S2-S3. 99 features were excluded from the nnU-Net model 535 Brown's method p-value calculation. 106 features were excluded from the RECOMIA Brown's method p-value calculation. All feature classes were affected. The most affected groups are Neighboring Gray Tone Difference Matrix (NGTDM), followed by Co-occurrence Matrix (GLCM) and first order. We have performed the bootstrap analysis and added the results accordingly. Generally, the ranking of segmentation algorithms remained unchanged following bootstrap analysis, which further supports the robustness of the findings.
- c) We acknowledge that the aim of these metrics is not to provide a direct biological or clinical interpretation of individual radiomics features. However, they serve as measures of overall agreement between segmentation methods across 107 radiomic features. We have clarified this in the Discussion section.
- Brown, M.B., *400: A Method for Combining Non-Independent, One-Sided Tests of Significance.* Biometrics, 1975. **31**(4): p. 987-992 DOI: 10.2307/2529826.

R4.6. Conventional segmentation metrics are underemphasized

The manuscript places strong emphasis on radiomics agreement while relatively downplaying classical segmentation metrics such as:

Dice similarity coefficient (DSC),

Hausdorff distance (HD),

surface-based metrics.

However, in clinical practice, accurate contour delineation remains critically important, especially for:

radiotherapy planning,

response assessment,

volumetric quantification.

Radiomics agreement should probably be presented as a complementary evaluation approach rather than a replacement for established segmentation metrics.

- **Agree – revised.** Thank you for this important comment. We acknowledge that this has been mentioned in the Discussion section "Ultimately, these results suggest that radiomics features, together with our proposed comparison method may serve as valuable complementary tools to conventional metrics for evaluating and comparing segmentation algorithms in clinical and research settings." We have also clarified this in the Conclusions section.

R4.7. Limited clinical relevance

The manuscript would benefit from a clearer discussion of the clinical implications of improved radiomics agreement.

At present, the study does not demonstrate whether higher radiomics agreement leads to improvements in:

prognosis prediction,

treatment response assessment,

radiotherapy planning accuracy,

or clinical outcomes.

Therefore, the practical clinical significance remains speculative.

- **Partially agree – revised:** Thank you for this important comment.
- We acknowledge that the present study was not designed to evaluate the prognosis prediction, treatment response assessment, radiotherapy planning accuracy or clinical outcomes. Rather, the objective of this study was to develop a complementary comparison method, based on radiomics features extracted from the segmentation outputs, which take into account the tumor shape, intensity, and texture characteristics, in addition to classical metrics when comparing segmentation algorithms. We have acknowledged this in the limitation paragraph of the Discussion section: "Firstly, we performed a plain methodological investigation, without any direct clinical correlation with lung cancer-related outcomes, such as diagnosis, treatment response and survival."

- While higher radiomics agreement does not directly demonstrate improved clinical outcomes, it suggests that a segmentation method could accurately delineate the tumor, which is important for many clinical applications. Therefore, it could significantly enhance clinical outcomes and allow accurate and efficient diagnosis and treatment planning, as demonstrated by Borrelli et al and Rayed et al. We have revised the Discussion section accordingly.
- Borrelli, P., et al., *Freely available convolutional neural network-based quantification of PET/CT lesions is associated with survival in patients with lung cancer.* EJNMMI Phys, 2022. **9**(1): p. 6 DOI: 10.1186/s40658-022-00437-3.
- Rayed, M.E., et al., *Deep learning for medical image segmentation: State-of-the-art advancements and challenges*. Informatics in Medicine Unlocked, 2024. **47**: p. 101504 DOI: 10.1016/j.imu.2024.101504.

R4.8. External validation is lacking

The study is based on a single-center retrospective dataset. PET radiomics and segmentation performance are highly scanner- and protocol-dependent.

External validation using independent datasets would substantially strengthen the conclusions.

- **Agree - revised.** Thank you for this important suggestion. We agree that external validation could improve the generalizability of our findings. We have acknowledged this in the limitation paragraph of the Discussion section: "We also did not conduct external prospective validation or detailed subgroup analyses (e.g., by scanner brands, lesion size), which could reveal different context-specific performance patterns."

Minor Comments

R4.9. Please clarify whether PET and CT channels were jointly used during segmentation inference in nnU-Net.

- **Agree – revised**: Thank you for this important comment. Both PET and CT images are used during training and testing by nnU-Net. We have revised the Algorithms section accordingly.

R4.10. The manuscript would benefit from a clearer explanation of why wavelet features were not included.

- **Agree – revised**: Thank you for this important comment. Wavelet and Laplacian-of-Gaussian features were not included because RECOMIA only provides us the extracted radiomics features but not the corresponding segmentation masks required to generate filtered radiomics features. To ensure a consistent comparison across all segmentation methods, the analysis was therefore restricted to the original set of radiomics features that are available for all methods. We have acknowledged this in the limitation paragraph of the Discussion section.

R4.11. Please provide additional details regarding failed segmentation cases and failure mechanisms.

- **Agree – revised:** Thank you for this important comment. We have provided additional details in the Algorithms section.

R4.12. Figures remain somewhat excessive despite revisions; further consolidation may improve readability.

- **Agree – revised:** Thank you for this important comment. We have consolidated Figure 4 and Figure 5 into 1 figure to improve readability.

R4.13. Some wording in the Discussion still implies causal superiority of nnU-Net beyond what the data support.

- **Agree – revised:** Thank you for this important comment. We have revised the Discussion and Conclusion sections accordingly.
