# Supplementary material for: Assessment of algorithmic performance in PET-CT segmentation of solitary lung tumors
Source: PLoS One. 2026 Nov 5;21(11):e0360849. doi: 10.1371/journal.pone.0360849 (PMC13739142; doi:10.1371/journal.pone.0360849)
Supplement: Supplementary file 3 [file pone.0360849.s001.docx]

**Table S1.** Feature-wise Wilcoxon p-values and correlations between RECOMIA, nnU-Net model 535 and manual mask’s radiomics features. P-values are shown before FDR and Bonferroni correction. Not available (NA) values were removed from the calculation of sums of absolute value of correlations.

| Radiomics parameter | *RECOMIA p-value* | *nnU-Net p-value* | *RECOMIA cor* | *nnU-Net cor* |
| --- | --- | --- | --- | --- |
| original_shape_Elongation | 0.780843174 | 0.642862216 | 0.594069421 | 0.802358725 |
| original_shape_Flatness | 0.504306616 | 0.752521391 | 0.594632292 | 0.735785405 |
| original_shape_LeastAxisLength | < 0.001 | 0.458135157 | 0.743847869 | 0.91024428 |
| original_shape_MajorAxisLength | < 0.001 | 0.480919136 | 0.270026777 | 0.874291898 |
| original_shape_Maximum2DDiameterColumn | < 0.001 | 0.425080042 | 0.754153608 | 0.943857673 |
| original_shape_Maximum2DDiameterRow | < 0.001 | 0.478023437 | 0.746759877 | 0.855712162 |
| original_shape_Maximum2DDiameterSlice | < 0.001 | 0.35368938 | 0.776833109 | 0.915615619 |
| original_shape_Maximum3DDiameter | < 0.001 | 0.555913407 | 0.509983306 | 0.886761047 |
| original_shape_MeshVolume | < 0.001 | 0.312079869 | 0.484979099 | 0.865055463 |
| original_shape_MinorAxisLength | < 0.001 | 0.458135157 | 0.781432331 | 0.932283428 |
| original_shape_Sphericity | < 0.001 | 0.831091173 | 0.316430151 | 0.494892456 |
| original_shape_SurfaceArea | < 0.001 | 0.403908489 | 0.756661393 | 0.939652597 |
| original_shape_SurfaceVolumeRatio | < 0.001 | 0.158643043 | 0.221912143 | 0.815411022 |
| original_shape_VoxelVolume | < 0.001 | 0.312079869 | 0.487441079 | 0.865158865 |
| original_firstorder_10Percentile | < 0.001 | 0.000642904 | -0.034201499 | 0.151841344 |
| original_firstorder_90Percentile | < 0.001 | 0.504306616 | -0.122785636 | 0.870147865 |
| original_firstorder_Energy | < 0.001 | 0.770184927 | -0.043949533 | 0.764587659 |
| original_firstorder_Entropy | < 0.001 | 0.874726118 | NA | 0.741663298 |
| original_firstorder_InterquartileRange | < 0.001 | 0.81665426 | -0.114414454 | 0.727109019 |
| original_firstorder_Kurtosis | < 0.001 | 0.738481822 | 0.047173585 | -0.018865937 |
| original_firstorder_Maximum | < 0.001 | 0.703771314 | -0.140962235 | 0.861449078 |
| original_firstorder_MeanAbsoluteDeviation | < 0.001 | 0.752521391 | -0.125690308 | 0.783543718 |
| original_firstorder_Mean | < 0.001 | 0.091436102 | -0.100104064 | 0.622542202 |
| original_firstorder_Median | < 0.001 | 0.020930858 | -0.083083135 | 0.522253645 |
| original_firstorder_Minimum | < 0.001 | < 0.001 | 0.05843486 | -0.007463477 |
| original_firstorder_Range | < 0.001 | 0.955625348 | -0.151790785 | 0.786229983 |
| original_firstorder_RobustMeanAbsoluteDeviation | < 0.001 | 0.795116503 | -0.118609079 | 0.739041166 |
| original_firstorder_RootMeanSquared | < 0.001 | 0.261796277 | -0.113918598 | 0.716747331 |
| original_firstorder_Skewness | < 0.001 | 0.312079869 | 0.036553273 | 0.354312203 |
| original_firstorder_TotalEnergy | < 0.001 | 0.770184927 | -0.043949533 | 0.764587659 |
| original_firstorder_Uniformity | < 0.001 | 0.838331388 | NA | 0.199093102 |
| original_firstorder_Variance | < 0.001 | 0.752521391 | -0.123318319 | 0.811925446 |
| original_glcm_Autocorrelation | < 0.001 | 0.911387843 | NA | 0.404089593 |
| original_glcm_ClusterProminence | < 0.001 | 0.773733777 | NA | 0.784888994 |
| original_glcm_ClusterShade | < 0.001 | 0.629629286 | NA | 0.576589602 |
| original_glcm_ClusterTendency | < 0.001 | 0.623056793 | NA | 0.773488905 |
| original_glcm_Contrast | < 0.001 | 0.368344176 | NA | 0.885805877 |
| original_glcm_Correlation | < 0.001 | 0.007992941 | NA | 0.00687901 |
| original_glcm_DifferenceAverage | < 0.001 | 0.277895272 | NA | 0.893741557 |
| original_glcm_DifferenceEntropy | < 0.001 | 0.435969626 | NA | 0.85943323 |
| original_glcm_DifferenceVariance | < 0.001 | 0.552821923 | NA | 0.659804646 |
| original_glcm_Id | < 0.001 | 0.194163012 | NA | 0.454932304 |
| original_glcm_Idm | < 0.001 | 0.153234857 | NA | 0.278614634 |
| original_glcm_Idmn | < 0.001 | 0.023644366 | NA | 0.69792655 |
| original_glcm_Idn | < 0.001 | 0.021981852 | NA | 0.575290853 |
| original_glcm_Imc1 | < 0.001 | 0.137850803 | NA | 0.897097089 |
| original_glcm_Imc2 | < 0.001 | 0.203892401 | NA | 0.816221479 |
| original_glcm_InverseVariance | < 0.001 | 0.158643043 | NA | 0.574340462 |
| original_glcm_JointAverage | < 0.001 | 0.795116503 | NA | 0.561934115 |
| original_glcm_JointEnergy | < 0.001 | 0.168450889 | NA | -0.090567005 |
| original_glcm_JointEntropy | < 0.001 | 0.603520351 | NA | 0.700861793 |
| original_glcm_MCC | < 0.001 | 0.006581972 | NA | 0.464473447 |
| original_glcm_MaximumProbability | < 0.001 | 0.040407683 | NA | -0.10047128 |
| original_glcm_SumAverage | < 0.001 | 0.795116503 | NA | 0.561934115 |
| original_glcm_SumEntropy | < 0.001 | 0.76664331 | NA | 0.673597058 |
| original_glcm_SumSquares | < 0.001 | 0.683232129 | NA | 0.786705118 |
| original_gldm_DependenceEntropy | < 0.001 | 0.172794291 | -0.169515061 | 0.791495212 |
| original_gldm_DependenceNonUniformity | < 0.001 | 0.534363508 | 0.194673593 | 0.952249514 |
| original_gldm_DependenceNonUniformityNormalized | < 0.001 | 0.158643043 | -0.175586208 | 0.581776918 |
| original_gldm_DependenceVariance | < 0.001 | 0.112771912 | -0.225659949 | -0.067223906 |
| original_gldm_GrayLevelNonUniformity | < 0.001 | 0.373301982 | 0.423846189 | 0.385808904 |
| original_gldm_GrayLevelVariance | < 0.001 | 0.752521391 | NA | 0.811921112 |
| original_gldm_HighGrayLevelEmphasis | < 0.001 | 0.904037831 | NA | 0.394957701 |
| original_gldm_LargeDependenceEmphasis | < 0.001 | 0.125964209 | -0.173814194 | -0.083289142 |
| original_gldm_LargeDependenceHighGrayLevelEmphasis | < 0.001 | 0.926110012 | 0.35939196 | 0.294709427 |
| original_gldm_LargeDependenceLowGrayLevelEmphasis | < 0.001 | 0.00481285 | 0.023915113 | -0.104129893 |
| original_gldm_LowGrayLevelEmphasis | < 0.001 | 0.036088664 | NA | 0.275120803 |
| original_gldm_SmallDependenceEmphasis | < 0.001 | 0.145387061 | -0.155620602 | 0.611717772 |
| original_gldm_SmallDependenceHighGrayLevelEmphasis | < 0.001 | 0.882039557 | -0.119521287 | 0.484044559 |
| original_gldm_SmallDependenceLowGrayLevelEmphasis | < 0.001 | 0.738481822 | 0.083903395 | 0.659027494 |
| original_glrlm_GrayLevelNonUniformity | 0.242594782 | 0.353718194 | 0.642206008 | 0.7144464 |
| original_glrlm_GrayLevelNonUniformityNormalized | < 0.001 | 0.896696031 | NA | 0.663094781 |
| original_glrlm_GrayLevelVariance | < 0.001 | 0.773733777 | NA | 0.820788332 |
| original_glrlm_HighGrayLevelRunEmphasis | < 0.001 | 0.940857526 | NA | 0.394135979 |
| original_glrlm_LongRunEmphasis | < 0.001 | 0.110683099 | -0.041414813 | -0.102066851 |
| original_glrlm_LongRunHighGrayLevelEmphasis | < 0.001 | 0.948239214 | 0.143281363 | 0.307330039 |
| original_glrlm_LongRunLowGrayLevelEmphasis | < 0.001 | 0.024812382 | -0.1011895 | 0.047714919 |
| original_glrlm_LowGrayLevelRunEmphasis | < 0.001 | 0.041322152 | NA | 0.379512445 |
| original_glrlm_RunEntropy | < 0.001 | 0.787970938 | 0.354418192 | 0.832629321 |
| original_glrlm_RunLengthNonUniformity | < 0.001 | 0.334796613 | 0.877666306 | 0.88792549 |
| original_glrlm_RunLengthNonUniformityNormalized | < 0.001 | 0.184778784 | -0.230899477 | 0.354654402 |
| original_glrlm_RunPercentage | < 0.001 | 0.132996372 | -0.260819934 | 0.028411435 |
| original_glrlm_RunVariance | < 0.001 | 0.071993596 | 0.061419186 | -0.113895003 |
| original_glrlm_ShortRunEmphasis | < 0.001 | 0.16702452 | -0.354291507 | 0.304803941 |
| original_glrlm_ShortRunHighGrayLevelEmphasis | < 0.001 | 0.940857526 | -0.319604281 | 0.400680664 |
| original_glrlm_ShortRunLowGrayLevelEmphasis | < 0.001 | 0.043203744 | 0.200478677 | 0.428685049 |
| original_glszm_GrayLevelNonUniformity | < 0.001 | 0.41975915 | 0.00962304 | 0.87742654 |
| original_glszm_GrayLevelNonUniformityNormalized | < 0.001 | 0.904037831 | NA | 0.923193786 |
| original_glszm_GrayLevelVariance | < 0.001 | 0.926110012 | NA | 0.846850985 |
| original_glszm_HighGrayLevelZoneEmphasis | < 0.001 | 0.904037831 | NA | 0.382086831 |
| original_glszm_LargeAreaEmphasis | < 0.001 | 0.102631809 | 0.129649459 | -0.029632005 |
| original_glszm_LargeAreaHighGrayLevelEmphasis | 0.368336358 | 0.486710058 | -0.078085629 | 0.270511462 |
| original_glszm_LargeAreaLowGrayLevelEmphasis | < 0.001 | 0.005715776 | 0.130748266 | -0.101875181 |
| original_glszm_LowGrayLevelZoneEmphasis | < 0.001 | 0.41975915 | NA | 0.70994473 |
| original_glszm_SizeZoneNonUniformity | < 0.001 | 0.623056793 | -0.045889035 | 0.935553267 |
| original_glszm_SizeZoneNonUniformityNormalized | < 0.001 | 0.14284069 | 0.012373877 | 0.794210933 |
| original_glszm_SmallAreaEmphasis | < 0.001 | 0.140328687 | 0.000498204 | 0.800338014 |
| original_glszm_SmallAreaHighGrayLevelEmphasis | < 0.001 | 0.948239214 | 0.313060575 | 0.387370309 |
| original_glszm_SmallAreaLowGrayLevelEmphasis | < 0.001 | 0.948239214 | -0.090926278 | 0.723997908 |
| original_glszm_ZoneEntropy | < 0.001 | 0.253993267 | 0.154666692 | 0.878424684 |
| original_glszm_ZonePercentage | < 0.001 | 0.153234857 | -0.166849362 | 0.552637832 |
| original_glszm_ZoneVariance | < 0.001 | 0.098784652 | -0.034401048 | -0.028420886 |
| original_ngtdm_Busyness | < 0.001 | 0.738481822 | NA | 0.747968095 |
| original_ngtdm_Coarseness | < 0.001 | 0.510245834 | NA | 0.876972966 |
| original_ngtdm_Complexity | < 0.001 | 0.303284473 | NA | 0.82608328 |
| original_ngtdm_Contrast | < 0.001 | 0.123685637 | NA | 0.871501345 |
| original_ngtdm_Strength | < 0.001 | 0.403908489 | NA | 0.803584435 |
