# Supplementary material for: Assessment of algorithmic performance in PET-CT segmentation of solitary lung tumors
Source: PLoS One. 2026 Nov 5;21(11):e0360849. doi: 10.1371/journal.pone.0360849 (PMC13739142; doi:10.1371/journal.pone.0360849)
Supplement: Supplementary file 4 [file pone.0360849.s002.docx]

**Table S2.** Feature-wise p-values used in calculation of nnU-Net model 535 Brown’s method combined p-value. For each radiomics parameter, the table reports Wilcoxon signed rank test p-values calculated using segmentation algorithm’s predicted masks and manual masks.

| Radiomics parameter | nnU-Net Wilcoxon p-value |
| --- | --- |
| original_shape_Elongation | 0.644569 |
| original_shape_Flatness | 0.753801 |
| original_shape_SurfaceVolumeRatio | 0.159432 |
| original_firstorder_Maximum | 0.705257 |
| original_glcm_DifferenceEntropy | 0.438002 |
| original_gldm_Dependen ceNonUniformityNormalized | 0.159432 |
| original_glrlm_RunEntropy | 0.789087 |
| original_glszm_ZoneEntropy | 0.255495 |
