# Supplementary material for: Assessment of algorithmic performance in PET-CT segmentation of solitary lung tumors
Source: PLoS One. 2026 Nov 5;21(11):e0360849. doi: 10.1371/journal.pone.0360849 (PMC13739142; doi:10.1371/journal.pone.0360849)
Supplement: Supplementary file 5 [file pone.0360849.s003.docx]

**Table S3.** Feature-wise p-values used in calculation of RECOMIA Brown’s method combined p-value. For each radiomics parameter, the table reports Wilcoxon signed rank test p-values calculated using segmentation algorithm’s predicted masks and manual masks.

| Radiomics parameter | RECOMIA Wilcoxon p-value |
| --- | --- |
| original_glrlm_RunEntropy | < 0.001 |
