# Supplementary material for: Assessment of algorithmic performance in PET-CT segmentation of solitary lung tumors
Source: PLoS One. 2026 Nov 5;21(11):e0360849. doi: 10.1371/journal.pone.0360849 (PMC13739142; doi:10.1371/journal.pone.0360849)
Supplement: Supplementary file 6 [file pone.0360849.s004.docx]

**Table S4.** Feature-wise correlations used in calculation of nnU-Net model 535 and RECOMIA sum of absolute values of correlation. For each radiomics parameter, the table reports correlation (cor) between segmentation algorithm’s predicted masks and manual masks**.**

| Radiomics parameter | NnU-Net cor | RECOMIA cor |
| --- | --- | --- |
| original_shape_Elongation | 0.802358725 | 0.594069421 |
| original_shape_Flatness | 0.735785405 | 0.594632292 |
| original_shape_LeastAxisLength | 0.91024428 | 0.743847869 |
| original_shape_MajorAxisLength | 0.874291898 | 0.270026777 |
| original_shape_Maximum2DDiameterColumn | 0.943857673 | 0.754153608 |
| original_shape_Maximum2DDiameterRow | 0.855712162 | 0.746759877 |
| original_shape_Maximum2DDiameterSlice | 0.915615619 | 0.776833109 |
| original_shape_Maximum3DDiameter | 0.886761047 | 0.509983306 |
| original_shape_MeshVolume | 0.865055463 | 0.484979099 |
| original_shape_MinorAxisLength | 0.932283428 | 0.781432331 |
| original_shape_Sphericity | 0.494892456 | 0.316430151 |
| original_shape_SurfaceArea | 0.939652597 | 0.756661393 |
| original_shape_SurfaceVolumeRatio | 0.815411022 | 0.221912143 |
| original_shape_VoxelVolume | 0.865158865 | 0.487441079 |
| original_firstorder_10Percentile | 0.151841344 | -0.034201499 |
| original_firstorder_90Percentile | 0.870147865 | -0.122785636 |
| original_firstorder_Energy | 0.764587659 | -0.043949533 |
| original_firstorder_InterquartileRange | 0.727109019 | -0.114414454 |
| original_firstorder_Kurtosis | -0.018865937 | 0.047173585 |
| original_firstorder_Maximum | 0.861449078 | -0.140962235 |
| original_firstorder_MeanAbsoluteDeviation | 0.783543718 | -0.125690308 |
| original_firstorder_Mean | 0.622542202 | -0.100104064 |
| original_firstorder_Median | 0.522253645 | -0.083083135 |
| original_firstorder_Minimum | -0.007463477 | 0.05843486 |
| original_firstorder_Range | 0.786229983 | -0.151790785 |
| original_firstorder_RobustMeanAbsoluteDeviation | 0.739041166 | -0.118609079 |
| original_firstorder_RootMeanSquared | 0.716747331 | -0.113918598 |
| original_firstorder_Skewness | 0.354312203 | 0.036553273 |
| original_firstorder_TotalEnergy | 0.764587659 | -0.043949533 |
| original_firstorder_Variance | 0.811925446 | -0.123318319 |
| original_gldm_DependenceEntropy | 0.791495212 | -0.169515061 |
| original_gldm_DependenceNonUniformity | 0.952249514 | 0.194673593 |
| original_gldm_DependenceNonUniformityNormalized | 0.581776918 | -0.175586208 |
| original_gldm_DependenceVariance | -0.067223906 | -0.225659949 |
| original_gldm_GrayLevelNonUniformity | 0.385808904 | 0.423846189 |
| original_gldm_LargeDependenceEmphasis | -0.083289142 | -0.173814194 |
| original_gldm_LargeDependenceHighGrayLevelEmphasis | 0.294709427 | 0.35939196 |
| original_gldm_LargeDependenceLowGrayLevelEmphasis | -0.104129893 | 0.023915113 |
| original_gldm_SmallDependenceEmphasis | 0.611717772 | -0.155620602 |
| original_gldm_SmallDependenceHighGrayLevelEmphasis | 0.484044559 | -0.119521287 |
| original_gldm_SmallDependenceLowGrayLevelEmphasis | 0.659027494 | 0.083903395 |
| original_glrlm_GrayLevelNonUniformity | 0.7144464 | 0.642206008 |
| original_glrlm_LongRunEmphasis | -0.102066851 | -0.041414813 |
| original_glrlm_LongRunHighGrayLevelEmphasis | 0.307330039 | 0.143281363 |
| original_glrlm_LongRunLowGrayLevelEmphasis | 0.047714919 | -0.1011895 |
| original_glrlm_RunEntropy | 0.832629321 | 0.354418192 |
| original_glrlm_RunLengthNonUniformity | 0.88792549 | 0.877666306 |
| original_glrlm_RunLengthNonUniformityNormalized | 0.354654402 | -0.230899477 |
| original_glrlm_RunPercentage | 0.028411435 | -0.260819934 |
| original_glrlm_RunVariance | -0.113895003 | 0.061419186 |
| original_glrlm_ShortRunEmphasis | 0.304803941 | -0.354291507 |
| original_glrlm_ShortRunHighGrayLevelEmphasis | 0.400680664 | -0.319604281 |
| original_glrlm_ShortRunLowGrayLevelEmphasis | 0.428685049 | 0.200478677 |
| original_glszm_GrayLevelNonUniformity | 0.87742654 | 0.00962304 |
| original_glszm_LargeAreaEmphasis | -0.029632005 | 0.129649459 |
| original_glszm_LargeAreaHighGrayLevelEmphasis | 0.270511462 | -0.078085629 |
| original_glszm_LargeAreaLowGrayLevelEmphasis | -0.101875181 | 0.130748266 |
| original_glszm_SizeZoneNonUniformity | 0.935553267 | -0.045889035 |
| original_glszm_SizeZoneNonUniformityNormalized | 0.794210933 | 0.012373877 |
| original_glszm_SmallAreaEmphasis | 0.800338014 | 0.000498204 |
| original_glszm_SmallAreaHighGrayLevelEmphasis | 0.387370309 | 0.313060575 |
| original_glszm_SmallAreaLowGrayLevelEmphasis | 0.723997908 | -0.090926278 |
| original_glszm_ZoneEntropy | 0.878424684 | 0.154666692 |
| original_glszm_ZonePercentage | 0.552637832 | -0.166849362 |
| original_glszm_ZoneVariance | -0.028420886 | -0.034401048 |
