# Supplementary material for: Assessment of algorithmic performance in PET-CT segmentation of solitary lung tumors
Source: PLoS One. 2026 Nov 5;21(11):e0360849. doi: 10.1371/journal.pone.0360849 (PMC13739142; doi:10.1371/journal.pone.0360849)
Supplement: Supplementary file 7 [file pone.0360849.s005.docx]

**Table S5.** Per-case (dis)similarity metrics between from nnU-Net model 535 and manual masks. Similarity metrics (higher is better): Jaccard index (Jaccard), Pearson correlation coefficient (Pearson), mutual information (MI), and normalized mutual information (NMI). Dissimilarity metrics (lower is better): Kullback-Leibler divergence (KLD), L1 norm.

| Patient | Similarity metrics | | | | Dissimilarity metrics | |
| --- | --- | --- | --- | --- | --- | --- |
|  | Jaccard | Pearson | MI | NMI | KLD | L1 norm |
| p00 | 0.710 | 0.843 | 0.001 | 1.634 | -0.002 | 603 |
| p01 | 0.382 | 0.614 | 0.009 | 1.299 | 6.353 | 29585 |
| p02 | 0.782 | 0.883 | 0.007 | 1.685 | 6.945 | 2910 |
| p03 | 0.779 | 0.882 | 0.003 | 1.697 | -0.004 | 1260 |
| p04 | 0.755 | 0.868 | 0.004 | 1.667 | 7.544 | 1820 |
| p05 | 0.786 | 0.886 | 0.007 | 1.696 | 6.906 | 3004 |
| p06 | 0.874 | 0.934 | 0.003 | 1.800 | 7.785 | 597 |
| p07 | 0.794 | 0.890 | 0.003 | 1.710 | 8.036 | 822 |
| p08 | 0.954 | 0.977 | 0.001 | 1.919 | 9.265 | 58 |
| p09 | 0.857 | 0.926 | 0.003 | 1.788 | 8.073 | 534 |
| p10 | 0.858 | 0.926 | 0.002 | 1.794 | -0.002 | 356 |
| p11 | 0.648 | 0.805 | 0.002 | 1.563 | 8.278 | 1927 |
| p12 | 0.684 | 0.826 | 0.002 | 1.597 | 8.432 | 1243 |
| p13 | 0.625 | 0.780 | 0.001 | 1.530 | 8.899 | 556 |
| p14 | 0.657 | 0.810 | 0.002 | 1.572 | 8.283 | 1613 |
| p15 | 0.891 | 0.943 | 0.002 | 1.822 | 8.125 | 421 |
| p16 | 0.928 | 0.963 | 0.015 | 1.864 | 6.113 | 2066 |
| p17 | 0.831 | 0.912 | 0.002 | 1.759 | 8.000 | 704 |
| p18 | 0.931 | 0.965 | 0.001 | 1.891 | 9.206 | 94 |
| p19 | 0.796 | 0.891 | 0.001 | 1.724 | 9.489 | 199 |
| p20 | 0.953 | 0.976 | 0.006 | 1.906 | 7.213 | 473 |
| p21 | 0.614 | 0.784 | 0.001 | 1.539 | 8.705 | 753 |
| p22 | 0.538 | 0.734 | 0.093 | 1.995 | 8.350 | 747 |
| p23 | 0.677 | 0.823 | 0.001 | 1.606 | 9.449 | 299 |
| p24 | 0.754 | 0.861 | 0.001 | 1.656 | 8.600 | 514 |
| p25 | 0.844 | 0.916 | 0.005 | 1.752 | 7.411 | 1390 |
| p26 | 0.922 | 0.960 | 0.196 | 2.000 | 7.110 | 141 |
| p27 | 0.792 | 0.885 | 0.002 | 1.697 | 8.324 | 560 |
| p28 | 0.790 | 0.889 | 0.001 | 1.719 | 8.987 | 342 |
| p29 | 0.224 | 0.473 | 0.001 | 1.188 | 8.594 | 7607 |
| p30 | 0.717 | 0.836 | 0.003 | 1.602 | 7.758 | 1451 |
| p31 | 0.682 | 0.817 | 0.003 | 1.572 | 7.584 | 2220 |
| p32 | 0.693 | 0.831 | 0.003 | 1.602 | 8.010 | 1943 |
| p33 | 0.140 | 0.245 | 0.000 | 1.090 | 8.684 | 3353 |
| p34 | 0.667 | 0.813 | 0.002 | 1.577 | 8.613 | 1011 |
| p35 | 0.675 | 0.821 | 0.002 | 1.592 | 8.464 | 1641 |
| p36 | 0.488 | 0.698 | 0.268 | 1.995 | -0.002 | 3525 |
| p37 | 0.377 | 0.549 | 0.003 | 1.267 | 7.100 | 7855 |
| p38 | 0.587 | 0.762 | 0.002 | 1.497 | 8.400 | 2099 |
| p39 | 0.691 | 0.829 | 0.001 | 1.605 | 8.860 | 754 |
| p40 | 0.630 | 0.778 | 0.004 | 1.507 | 7.354 | 4000 |
| p41 | 0.598 | 0.758 | 0.005 | 1.475 | 7.026 | 5560 |
| p42 | 0.469 | 0.684 | 0.002 | 1.397 | -0.004 | 3614 |
| **Average** | **0.699** | **0.820** | **0.016** | **1.648** | **7.124** | **2377.302** |
