# Supplementary material for: Assessment of algorithmic performance in PET-CT segmentation of solitary lung tumors
Source: PLoS One. 2026 Nov 5;21(11):e0360849. doi: 10.1371/journal.pone.0360849 (PMC13739142; doi:10.1371/journal.pone.0360849)
Supplement: Supplementary file 8 [file pone.0360849.s006.docx]

**Table S6.** Per-case (dis)similarity metrics calculated from nnU-Net model 502 and manual masks. Similarity metrics (higher is better): Jaccard index (Jaccard), Pearson correlation coefficient (Pearson), mutual information (MI), and normalized mutual information (NMI). Dissimilarity metrics (lower is better): Kullback-Leibler divergence (KLD), L1 norm. Not available (N/A) values were removed from the calculation of average.

| Patient | Similarity metrics | | | | Dissimilarity metrics | |  |
| --- | --- | --- | --- | --- | --- | --- | --- |
|  | Jaccard | Pearson | MI | NMI | KLD | L1 norm | Note |
| p09 | 0.954 | 0.977 | 0.338 | 2.000 | 4.440 | 177 |  |
| p10 | 0.835 | 0.910 | 0.365 | 2.000 | 3.677 | 462 |  |
| p11 | 0.000 | N/A | 0.355 | 1.996 | -0.001 | 5467 | no mask |
| p12 | 0.810 | 0.896 | 0.356 | 1.999 | 3.956 | 787 |  |
| p13 | 0.620 | 0.777 | 0.337 | 1.999 | 4.967 | 568 |  |
| p14 | 0.682 | 0.811 | 0.356 | 1.998 | 3.844 | 1749 |  |
| p15 | 0.832 | 0.912 | 0.365 | 1.999 | 3.476 | 716 |  |
| p16 | 0.000 | N/A | 0.355 | 1.984 | -0.005 | 28435 | no mask |
| p17 | 0.742 | 0.861 | 0.365 | 1.999 | 3.436 | 1203 |  |
| p18 | 0.824 | 0.908 | 0.337 | 2.000 | 4.925 | 270 |  |
| p19 | 0.000 | N/A | 0.355 | 1.999 | 0.000 | 785 | no mask |
| p20 | 0.819 | 0.904 | 0.357 | 1.998 | 3.402 | 2141 |  |
| p21 | 0.061 | 0.248 | 0.356 | 1.989 | 3.164 | 18313 |  |
| p22 | 0.085 | 0.292 | 0.337 | 1.993 | 3.921 | 9338 |  |
| p23 | 0.475 | 0.689 | 0.356 | 1.999 | 4.424 | 694 |  |
| p24 | 0.606 | 0.775 | 0.356 | 1.999 | 4.083 | 1093 |  |
| p25 | 0.000 | N/A | 0.364 | 1.995 | -0.002 | 8583 | no mask |
| p26 | 0.928 | 0.963 | 0.356 | 2.000 | 4.291 | 128 |  |
| p27 | 0.000 | 0.000 | 0.355 | 1.994 | 3.683 | 8696 |  |
| p28 | 0.494 | 0.703 | 0.337 | 1.999 | 4.645 | 1315 |  |
| p29 | 0.000 | N/A | 0.337 | 1.993 | -0.002 | 9804 | no mask |
| p30 | 0.711 | 0.831 | 0.356 | 1.999 | 3.859 | 1453 |  |
| p31 | 0.723 | 0.846 | 0.356 | 1.999 | 3.911 | 1479 |  |
| p32 | 0.768 | 0.873 | 0.338 | 1.998 | 4.274 | 1508 |  |
| p33 | 0.000 | 0.000 | 0.337 | 1.994 | 4.268 | 7664 | no mask |
| p34 | 0.744 | 0.860 | 0.356 | 1.999 | 4.155 | 781 |  |
| p35 | 0.594 | 0.745 | 0.356 | 1.998 | 3.828 | 2485 |  |
| p36 | 0.570 | 0.754 | 0.338 | 1.997 | 4.423 | 2965 |  |
| p37 | 0.297 | 0.471 | 0.338 | 1.990 | 3.798 | 11262 |  |
| p38 | 0.000 | 0.000 | 0.364 | 1.997 | 5.126 | 5074 |  |
| p39 | 0.124 | 0.293 | 0.356 | 1.992 | 3.405 | 10937 |  |
| p40 | 0.797 | 0.890 | 0.339 | 1.998 | 4.029 | 2040 |  |
| p41 | 0.644 | 0.790 | 0.357 | 1.996 | 3.483 | 4897 |  |
| p42 | 0.556 | 0.746 | 0.356 | 1.997 | -0.001 | 3019 |  |
| **Average** | **0.479** | **0.680** | **0.351** | **1.997** | **3.320** | **4596** |  |
