# Supplementary material for: Assessment of algorithmic performance in PET-CT segmentation of solitary lung tumors
Source: PLoS One. 2026 Nov 5;21(11):e0360849. doi: 10.1371/journal.pone.0360849 (PMC13739142; doi:10.1371/journal.pone.0360849)
Supplement: Supplementary file 9 [file pone.0360849.s007.docx]

**Table S7.** Per-case (dis)similarity metrics calculated from nnU-Net model 503 and manual masks. Similarity metrics (higher is better): Jaccard index (Jaccard), Pearson correlation coefficient (Pearson), mutual information (MI), and normalized mutual information (NMI). Dissimilarity metrics (lower is better): Kullback-Leibler divergence (KLD), L1 norm. Not available (N/A) values were removed from the calculation of average.

| Patient | Similarity metrics | | | | Dissimilarity metrics | |  |
| --- | --- | --- | --- | --- | --- | --- | --- |
|  | Jaccard | Pearson | MI | NMI | KLD | L1 norm | Note |
| p00 | 0.351 | 0.593 | 0.356 | 1.999 | -0.001 | 1350 |  |
| p01 | 0.000 | N/A | 0.355 | 1.976 | -0.008 | 47568 | no mask |
| p02 | 0.000 | N/A | 0.355 | 1.992 | -0.003 | 13218 | no mask |
| p03 | 0.770 | 0.870 | 0.365 | 1.999 | 3.377 | 1435 |  |
| p04 | 0.000 | N/A | 0.337 | 1.994 | -0.002 | 7407 | no mask |
| p05 | 0.000 | N/A | 0.355 | 1.991 | -0.003 | 14015 | no mask |
| p06 | 0.863 | 0.929 | 0.338 | 1.999 | 4.452 | 584 |  |
| p07 | 0.774 | 0.879 | 0.356 | 1.999 | 3.795 | 1150 |  |
| p08 | 0.751 | 0.867 | 0.356 | 2.000 | 0.000 | 310 |  |
| p09 | 0.852 | 0.920 | 0.338 | 1.999 | 4.461 | 593 |  |
| p10 | 0.640 | 0.800 | 0.365 | 1.999 | -0.001 | 902 |  |
| p11 | 0.000 | N/A | 0.355 | 1.996 | -0.001 | 5467 | no mask |
| p16 | 0.000 | N/A | 0.355 | 1.984 | -0.005 | 28435 | no mask |
| p19 | 0.532 | 0.729 | 0.355 | 2.000 | 4.962 | 368 |  |
| p25 | 0.000 | N/A | 0.364 | 1.995 | -0.002 | 8583 | no mask |
| p29 | 0.077 | 0.278 | 0.337 | 1.993 | -0.002 | 9049 |  |
| p33 | 0.221 | 0.413 | 0.337 | 1.995 | 4.135 | 5853 | 2 masks |
| p38 | 0.007 | 0.086 | 0.364 | 1.997 | 0.000 | 4954 |  |
| p39 | 0.451 | 0.671 | 0.356 | 1.999 | 4.513 | 1324 |  |
| p40 | 0.673 | 0.819 | 0.338 | 1.997 | 4.138 | 3229 |  |
| p41 | 0.874 | 0.933 | 0.358 | 1.998 | 3.388 | 1674 |  |
| p42 | 0.419 | 0.647 | 0.356 | 1.997 | -0.001 | 3950 |  |
| **Average** | **0.383** | **0.716** | **0.353** | **1.995** | **1.574** | **7407** |  |
