# Supplementary material for: Assessment of algorithmic performance in PET-CT segmentation of solitary lung tumors
Source: PLoS One. 2026 Nov 5;21(11):e0360849. doi: 10.1371/journal.pone.0360849 (PMC13739142; doi:10.1371/journal.pone.0360849)
Supplement: Supplementary file 10 [file pone.0360849.s008.docx]

**Table S8.** Dice Similarity Coefficients (DSC) and Hausdorff Distances (HD) between nnU-Net models 502, 503, 535 and manual masks. Failed cases (no mask or double masks) were removed from the calculation. NM = no mask, DM = double masks and TR: used for training.

| Patient | **DSC** | | | **Hausdorff Distance** | | |
| --- | --- | --- | --- | --- | --- | --- |
|  | **n502** | **n503** | **n535** | **n502** | **n503** | **n535** |
| p000 | TR | 0.50 | 0.83 | TR | 3.89 | 1.83 |
| p001 | TR | NM | 0.55 | TR | NM | 5.53 |
| p002 | TR | NM | 0.88 | TR | NM | 2.04 |
| p003 | TR | 0.87 | 0.87 | TR | 1.90 | 1.76 |
| p004 | TR | NM | 0.86 | TR | NM | 1.93 |
| p005 | TR | NM | 0.35 | TR | NM | 1.05 |
| p006 | TR | 0.93 | 0.93 | TR | 1.37 | 1.53 |
| p007 | TR | 0.87 | 0.34 | TR | 1.71 | 0.56 |
| p008 | TR | 0.84 | 0.98 | TR | 1.58 | 0.57 |
| p009 | 0.98 | 0.91 | 0.91 | 0.65 | 1.50 | 1.47 |
| p010 | 0.95 | 0.78 | 0.91 | 0.95 | 2.47 | 1.62 |
| p011 | NM | NM | 0.79 | NM | NM | 2.67 |
| p012 | 0.89 | TR | 0.81 | 1.83 | TR | 2.47 |
| p013 | 0.77 | TR | 0.78 | 2.21 | TR | 2.05 |
| p014 | 0.88 | TR | 0.79 | 1.94 | TR | 2.77 |
| p015 | 0.91 | TR | 0.93 | 1.55 | TR | 1.03 |
| p016 | NM | NM | 0.96 | NM | NM | 1.49 |
| p017 | 0.85 | TR | 0.90 | 1.90 | TR | 1.67 |
| p018 | 0.90 | TR | 0.97 | 1.65 | TR | 0.95 |
| p019 | NM | 0.69 | 0.90 | NM | 2.31 | 1.22 |
| p020 | 0.90 | TR | 0.98 | 1.77 | TR | 0.67 |
| p021 | 0.00 | TR | 0.76 | 46.94 | TR | 2.07 |
| p022 | 0.00 | TR | 0.70 | 27.53 | TR | 2.65 |
| p023 | 0.64 | TR | 0.80 | 2.82 | TR | 1.84 |
| p024 | 0.75 | TR | 0.87 | 2.54 | TR | 1.73 |
| p025 | NM | NM | 0.92 | NM | NM | 1.52 |
| p026 | 0.96 | TR | 0.96 | 0.68 | TR | 0.73 |
| p027 | 0.00 | TR | 0.88 | 234.88 | TR | 1.70 |
| p028 | 0.91 | TR | 0.88 | 1.57 | TR | 1.52 |
| p029 | NM | 0.14 | 0.35 | NM | 15.88 | 9.20 |
| p030 | 0.83 | TR | 0.84 | 2.32 | TR | 2.50 |
| p031 | 0.84 | TR | 0.88 | 2.11 | TR | 1.76 |
| p032 | 0.87 | TR | 0.82 | 2.10 | TR | 2.67 |
| p033 | NM | DM | 0.24 | NM | DM | 8.00 |
| p034 | 0.86 | TR | 0.81 | 1.91 | TR | 2.32 |
| p035 | 0.00 | TR | 0.81 | 60.38 | TR | 2.57 |
| p036 | 0.73 | TR | 0.66 | 3.66 | TR | 4.46 |
| p037 | 0.46 | TR | 0.55 | 13.62 | TR | 9.97 |
| p038 | 0.00 | 0.01 | 0.75 | 280.24 | 17.73 | 3.27 |
| p039 | 0.00 | 0.62 | 0.81 | 139.36 | 3.65 | 2.09 |
| p040 | 0.89 | 0.81 | 0.78 | 2.22 | 3.16 | 5.35 |
| p041 | 0.79 | 0.93 | 0.75 | 4.35 | 1.64 | 4.83 |
| p042 | 0.71 | 0.59 | 0.63 | 3.88 | 5.45 | 4.83 |
| **Average** | **0.653** | **0.678** | **0.783** | **30.270** | **4.589** | **2.662** |
