# Supplementary material for: Assessment of algorithmic performance in PET-CT segmentation of solitary lung tumors
Source: PLoS One. 2026 Nov 5;21(11):e0360849. doi: 10.1371/journal.pone.0360849 (PMC13739142; doi:10.1371/journal.pone.0360849)
Supplement: Peer review history

# Peer review: Decision Letter 0

**DOI:** 10.1371/journal.pone.0360849.r001

**Authors:** Carmelo Caldarella

3 May 2026

Dear Dr. Le,

Thank you for submitting your manuscript to PLOS ONE. After careful consideration, we feel that it has merit but does not fully meet PLOS ONE’s publication criteria as it currently stands. Therefore, we invite you to submit a revised version of the manuscript that addresses the points raised during the review process.

==============================

**ACADEMIC EDITOR:**

==============================

Please submit your revised manuscript by Jun 17 2026 11:59PM. If you will need more time than this to complete your revisions, please reply to this message or contact the journal office at plosone@plos.org. When you're ready to submit your revision, log on to https://www.editorialmanager.com/pone/ and select the 'Submissions Needing Revision' folder to locate your manuscript file.

- A letter that responds to each point raised by the academic editor and reviewer(s). You should upload this letter as a separate file labeled 'Response to Reviewers'.
- A marked-up copy of your manuscript that highlights changes made to the original version. You should upload this as a separate file labeled 'Revised Manuscript with Track Changes'.
- An unmarked version of your revised paper without tracked changes. You should upload this as a separate file labeled 'Manuscript'.

If you would like to make changes to your financial disclosure, please include your updated statement in your cover letter. Guidelines for resubmitting your figure files are available below the reviewer comments at the end of this letter.

If applicable, we recommend that you deposit your laboratory protocols in protocols.io to enhance the reproducibility of your results. Protocols.io assigns your protocol its own identifier (DOI) so that it can be cited independently in the future. For instructions see: https://journals.plos.org/plosone/s/submission-guidelines#loc-laboratory-protocols. Additionally, PLOS ONE offers an option for publishing peer-reviewed Lab Protocol articles, which describe protocols hosted on protocols.io. Read more information on sharing protocols at https://plos.org/protocols?utm_medium=editorial-email&utm_source=authorletters&utm_campaign=protocols.

As the corresponding author, your ORCID iD is verified in the submission system and will appear in the published article. PLOS supports the use of ORCID, and we encourage all coauthors to register for an ORCID iD and use it as well. Please encourage your coauthors to verify their ORCID iD within the submission system before final acceptance, as unverified ORCID iDs will not appear in the published article. *Only*  the individual author can complete the verification step; PLOS staff *cannot*  verify ORCID iDs on behalf of authors.

We look forward to receiving your revised manuscript.

Kind regards,

Carmelo Caldarella, Ph.D., M.D.

Academic Editor

PLOS One

Journal Requirements:

When submitting your revision, we need you to address these additional requirements.

1. Please ensure that your manuscript meets PLOS ONE's style requirements, including those for file naming. The PLOS ONE style templates can be found at

https://journals.plos.org/plosone/s/file?id=wjVg/PLOSOne_formatting_sample_main_body.pdf and

https://journals.plos.org/plosone/s/file?id=ba62/PLOSOne_formatting_sample_title_authors_affiliations.pdf

2. The ethical approval number(s) listed in the manuscript and/or submission metadata does not match the approval number on the ethical approval document you provided. Please ensure that all approval numbers are correct.

3. Please note that PLOS One has specific guidelines on code sharing for submissions in which author-generated code underpins the findings in the manuscript. In these cases, we expect all author-generated code to be made available without restrictions upon publication of the work. Please review our guidelines at https://journals.plos.org/plosone/s/materials-and-software-sharing#loc-sharing-code and ensure that your code is shared in a way that follows best practice and facilitates reproducibility and reuse.

4. Thank you for stating the following in the Acknowledgments Section of your manuscript:

“Project no. TKP2021-NKTA-34 has been implemented with the support provided by the Ministry of Culture and Innovation of Hungary from the National Research, Development, and Innovation Fund, financed under the TKP2021-NKTA funding scheme.”

We note that you have provided funding information that is not currently declared in your Funding Statement. However, funding information should not appear in the Acknowledgments section or other areas of your manuscript. We will only publish funding information present in the Funding Statement section of the online submission form.

Please remove any funding-related text from the manuscript and let us know how you would like to update your Funding Statement. Currently, your Funding Statement reads as follows:

“Project no. TKP2021-NKTA-34 has been implemented with the support provided by the Ministry of Culture and Innovation of Hungary from the National Research, Development, and Innovation Fund, financed under the TKP2021-NKTA funding scheme.”

Please include your amended statements within your cover letter; we will change the online submission form on your behalf.

5. Thank you for stating the following financial disclosure:

“Project no. TKP2021-NKTA-34 has been implemented with the support provided by the Ministry of Culture and Innovation of Hungary from the National Research, Development, and Innovation Fund, financed under the TKP2021-NKTA funding scheme.”

Please state what role the funders took in the study.  If the funders had no role, please state: "The funders had no role in study design, data collection and analysis, decision to publish, or preparation of the manuscript."

If this statement is not correct you must amend it as needed.

Please include this amended Role of Funder statement in your cover letter; we will change the online submission form on your behalf.

6. In the online submission form you indicate that your data is not available for proprietary reasons and have provided a contact point for accessing this data. Please note that your current contact point is a co-author on this manuscript. According to our Data Policy, the contact point must not be an author on the manuscript and must be an institutional contact, ideally not an individual. Please revise your data statement to a non-author institutional point of contact, such as a data access or ethics committee, and send this to us via return email. Please also include contact information for the third party organization, and please include the full citation of where the data can be found.

7. Thank you for stating the following in the Competing Interests section:

The authors declare that they have no competing interests.

We note that one or more of the authors are employed by a commercial company: “Scanomed Ltd., Debrecen, Hungary”

1. Please provide an amended Funding Statement declaring this commercial affiliation, as well as a statement regarding the Role of Funders in your study. If the funding organization did not play a role in the study design, data collection and analysis, decision to publish, or preparation of the manuscript and only provided financial support in the form of authors' salaries and/or research materials, please review your statements relating to the author contributions, and ensure you have specifically and accurately indicated the role(s) that these authors had in your study. You can update author roles in the Author Contributions section of the online submission form.

Please also include the following statement within your amended Funding Statement.

“The funder provided support in the form of salaries for authors [insert relevant initials], but did not have any additional role in the study design, data collection and analysis, decision to publish, or preparation of the manuscript. The specific roles of these authors are articulated in the ‘author contributions’ section.”

If your commercial affiliation did play a role in your study, please state and explain this role within your updated Funding Statement.

2. Please also provide an updated Competing Interests Statement declaring this commercial affiliation along with any other relevant declarations relating to employment, consultancy, patents, products in development, or marketed products, etc.

Within your Competing Interests Statement, please confirm that this commercial affiliation does not alter your adherence to all PLOS ONE policies on sharing data and materials by including the following statement: "This does not alter our adherence to  PLOS ONE policies on sharing data and materials.” (as detailed online in our guide for authors http://journals.plos.org/plosone/s/competing-interests) . If this adherence statement is not accurate and  there are restrictions on sharing of data and/or materials, please state these. Please note that we cannot proceed with consideration of your article until this information has been declared.

Please include both an updated Funding Statement and Competing Interests Statement in your cover letter. We will change the online submission form on your behalf.

7. Your ethics statement should only appear in the Methods section of your manuscript. If your ethics statement is written in any section besides the Methods, please delete it from any other section.

8. We notice that your supplementary tables are included in the manuscript file. Please remove them and upload them with the file type 'Supporting Information'. Please ensure that each Supporting Information file has a legend listed in the manuscript after the references list.

9. If the reviewer comments include a recommendation to cite specific previously published works, please review and evaluate these publications to determine whether they are relevant and should be cited. There is no requirement to cite these works unless the editor has indicated otherwise.

Additional Editor Comments:

Dear Authors, please revise the paper according to the suggestions provided by the Reviewers, particularly explaining the clinical implications and usefulness of your results, and clarifying the real aim of the paper (to assess radiomic reproducibility or to find the most accurate segmentation software?). Moreover, provide more detailed discussion on radiomic parameters.

Best regards

Carmelo Caldarella

[Note: HTML markup is below. Please do not edit.]

Reviewers' comments:

Reviewer's Responses to Questions

**Comments to the Author**

1. Is the manuscript technically sound, and do the data support the conclusions?

Reviewer #1: Partly

Reviewer #2: Yes

Reviewer #3: Yes

**********

2. Has the statistical analysis been performed appropriately and rigorously?

Reviewer #1: I Don't Know

Reviewer #2: Yes

Reviewer #3: Yes

**********

3. Have the authors made all data underlying the findings in their manuscript fully available?

The PLOS Data policy

Reviewer #1: Yes

Reviewer #2: Yes

Reviewer #3: Yes

**********

4. Is the manuscript presented in an intelligible fashion and written in standard English?

Reviewer #1: Yes

Reviewer #2: Yes

Reviewer #3: Yes

**********

Reviewer #1: This is a paper evaluating different segmentation methods. From my point of view, it is not clear whether the aim is to assess radiomic reproducibility or to find the most accurate segmentation software. Here are my further comments:

“accurate diagnosis requires careful examination of PET-CT images by a skilled nuclear medicine expert, which is not only tedious but also extremely time-consuming”. I do not agree, since personally I need 10-15 min for lung cancer PET reporting assessment. The above-mentioned period can be true when dealing with contrast-enhanced PET/CT or tumor segmentation or response assessment based on standardized criteria.

“radiomic features offer a comprehensive characterization of the entire tumor, which is usually heterogeneous”. I would rather say that “radiomic CAN offer”

“Currently, there is a scarcity of published literature using radiomics features to compare the performance of different tumor segmentation algorithms on PET-CT images”. I hope that this is wrong since it seems that you segment tumor based on radiomic that is completely not clinically acceptable due to missing, strongly validated parameters. I think that the authors wanted to assess whether different segmentations system can obtain the same rame radiomic values.

In the whole paper, use proper radiopharmaceutical nomenclature according to EANM

Reduce the high number of figures by moving some to supplements.

From the results section, it is not clear which one was the more precise segmentation compared to the manual one, as in my eyes (DSC), it is a more important parameter than the radiomic one.

The results section is more similar to a table index, the authors should start presenting the main results of the whole paper.

“This study demonstrated that nnU-Net significantly outperforms RECOMIA in terms of PET-CT solitary lung tumor segmentation, as evidenced by closer agreement of radiomic features with manual reference masks”. In my eyes, the fact that radiomic is more reproducible il much more less important than the segmentation volume DSC. This should be consider by the authors who should modify the paper structure/results presentation accordingly. Otherwise, the study would focus only on radiomic features reproducibility among different segmentation software, which, however, cannot be considered to assess a segmentation software.

Reviewer #2: This is a well-designed and executed study to compare two recently developed AI-based autosegmentation tools. The analysis is very comprehensive, and the results are consistent with the conclusions. I have only a few comments as shown below.

General comments

1. A table summarizing the number of cases used for training and testing of two algorithms will help readers to understand the method more easily.

2. Radiomics features are used for performance comparison. However, the discussion of the results on radiomics is missing.

3. Furthermore, the introduction implies that radiomics application is main aim of this study by evidenced by one paragraph devoted to this topic. The first sentence of the last paragraph in Introduction gives the readers a false impression about the primary goal of this paper.

4. Please add more details about the radiomics feature calculations. What kind of filters were applied? Some publications show that the wavelet-based radiomics features are more sensitive to segmentation differences. See, for example, the following reference:

Akramova, R. and Y. Watanabe, Radiomics as a measure superior to common similarity metrics for tumor segmentation performance evaluation. J Appl Clin Med Phys, 2024. 25(8): p. e14442.

Reviewer #3: In this paper, the authors compared the performance of two deep learning-based algorithms (mnU-Net and RECOMIA) in the segmentation of solitary lung tumor at PET images after the generation of radiomic features, these latter able to better characterize lung tumor biology.

After comparative tests, the authors conclude that radiomic features generated by mnU-Net are more similar to those derived by manual segmentation, suggesting a superiority of this segmentation algorithm.

This manuscript is a pure methodological paper and no clinical correlation with radiomics data and features of lung cancer are provided. Which is the clinical impact of a better segmentation? Which parameters may be generated by an optimal segmentation? How it may be useful in diagnosis and monitoring of lung cancer?

Was the study retrospective or prospective? Please clarify

The projections reported in Figures 4-11 for two methods of analysis are often different. Please choose the three spatial projections for the two methods.

**********

what does this mean?). If published, this will include your full peer review and any attached files.

If you choose “no”, your identity will remain anonymous but your review may still be made public.

**Do you want your identity to be public for this peer review?**  For information about this choice, including consent withdrawal, please see our Privacy Policy

Reviewer #1: **Yes:**  Riccardo Laudicella

Reviewer #2: No

Reviewer #3: No

**********

[NOTE: If reviewer comments were submitted as an attachment file, they will be attached to this email and accessible via the submission site. Please log into your account, locate the manuscript record, and check for the action link "View Attachments". If this link does not appear, there are no attachment files.]

To ensure your figures meet our technical requirements, please review our figure guidelines: https://journals.plos.org/plosone/s/figures

You may also use PLOS’s free figure tool, NAAS, to help you prepare publication quality figures: https://journals.plos.org/plosone/s/figures#loc-tools-for-figure-preparation.

NAAS will assess whether your figures meet our technical requirements by comparing each figure against our figure specifications.

# Author comment: Author response to Decision Letter 1

**DOI:** 10.1371/journal.pone.0360849.r002

25 May 2026

Assessment of Algorithmic Performance in PET-CT Segmentation of Solitary Lung Tumors

Response to Reviewers

Academic Editor

Journal Requirements:

When submitting your revision, we need you to address these additional requirements.

E.1. Please ensure that your manuscript meets PLOS ONE's style requirements, including those for file naming. The PLOS ONE style templates can be found at

https://journals.plos.org/plosone/s/file?id=wjVg/PLOSOne_formatting_sample_main_body.pdf and https://journals.plos.org/plosone/s/file?id=ba62/PLOSOne_formatting_sample_title_authors_affiliations.pdf

• Agree – revised: Thank you for this important suggestion. We have revised the manuscript accordingly.

E.2. The ethical approval number(s) listed in the manuscript and/or submission metadata does not match the approval number on the ethical approval document you provided. Please ensure that all approval numbers are correct.

• Agree – revised: Thank you for this important comment. This is the correct Protocol ID (DE RKEB/IKEB-nél) 6504-2023, 13th September 2023). We have revised the manuscript accordingly.

E.3. Please note that PLOS One has specific guidelines on code sharing for submissions in which author-generated code underpins the findings in the manuscript. In these cases, we expect all author-generated code to be made available without restrictions upon publication of the work. Please review our guidelines at https://journals.plos.org/plosone/s/materials-and-software-sharing#loc-sharing-code and ensure that your code is shared in a way that follows best practice and facilitates reproducibility and reuse.

• Agree – revised: Thank you for this important comment. The codes are available at https://github.com/opgabor/nnUNet-vs-RECOMIA. We have requested for correction in the cover letter and sent you an Updated Data statement via email.

• Updated Data statement: “The data analyzed in this study, including sensitive patient information, will be made available upon reasonable request from the University of Debrecen Data Protection Officer in accordance with applicable ethical and data protection regulations. Address: University of Debrecen Data Protection Officer, Kassai út campus, H-4028 Debrecen, Kassai u. 26. Chancellery IV, ground floor 34, website: https://unideb.hu/en/data-protection-officer-dpo, email: adatvedelmi.tisztviselo@unideb.hu, phone: +36 52,512,700/74465. The source codes are available at https://github.com/opgabor/nnUNet-vs-RECOMIA.”

E.4. Thank you for stating the following in the Acknowledgments Section of your manuscript:

“Project no. TKP2021-NKTA-34 has been implemented with the support provided by the Ministry of Culture and Innovation of Hungary from the National Research, Development, and Innovation Fund, financed under the TKP2021-NKTA funding scheme.”

We note that you have provided funding information that is not currently declared in your Funding Statement. However, funding information should not appear in the Acknowledgments section or other areas of your manuscript. We will only publish funding information present in the Funding Statement section of the online submission form.

Please remove any funding-related text from the manuscript and let us know how you would like to update your Funding Statement. Currently, your Funding Statement reads as follows:

“Project no. TKP2021-NKTA-34 has been implemented with the support provided by the Ministry of Culture and Innovation of Hungary from the National Research, Development, and Innovation Fund, financed under the TKP2021-NKTA funding scheme.”

Please include your amended statements within your cover letter; we will change the online submission form on your behalf.

• Agree – revised: Thank you for this important comment. We have deleted the funding from the Acknowledgments section. We have included the updated Funding statement in our cover letter.

E.5. Thank you for stating the following financial disclosure:

“Project no. TKP2021-NKTA-34 has been implemented with the support provided by the Ministry of Culture and Innovation of Hungary from the National Research, Development, and Innovation Fund, financed under the TKP2021-NKTA funding scheme.”

Please state what role the funders took in the study. If the funders had no role, please state: "The funders had no role in study design, data collection and analysis, decision to publish, or preparation of the manuscript."

If this statement is not correct you must amend it as needed.

Please include this amended Role of Funder statement in your cover letter; we will change the online submission form on your behalf.

• Agree – revised: Thank you for this important comment. The mentioned sentence belongs to the Funding statement, not the financial disclosure. I am sorry for this misunderstanding. The request for correction has been included in the cover letter.

• Updated Financial Disclosure Statement “Project no. TKP2021-NKTA-34 has been implemented with the support provided by the Ministry of Culture and Innovation of Hungary from the National Research, Development, and Innovation Fund, financed under the TKP2021-NKTA funding scheme. The funders had no role in study design, data collection and analysis, decision to publish, or preparation of the manuscript. Scanomed Ltd., provided support in the form of salaries for authors Márton Mikó, Bence Farkas, but did not have any additional role in the study design, data collection and analysis, decision to publish, or preparation of the manuscript. The specific roles of these authors are articulated in the ‘author contributions’ section.”

E.6. In the online submission form you indicate that your data is not available for proprietary reasons and have provided a contact point for accessing this data. Please note that your current contact point is a co-author on this manuscript. According to our Data Policy, the contact point must not be an author on the manuscript and must be an institutional contact, ideally not an individual. Please revise your data statement to a non-author institutional point of contact, such as a data access or ethics committee, and send this to us via return email. Please also include contact information for the third party organization, and please include the full citation of where the data can be found.

• Agree – revised: Thank you for this important comment. The request for correction has been included in the cover letter and the updated Data statement has been sent to you through return email.

• Updated Data statement: “The data analyzed in this study, including sensitive patient information, will be made available upon reasonable request from the University of Debrecen Data Protection Officer in accordance with applicable ethical and data protection regulations. Address: University of Debrecen Data Protection Officer, Kassai út campus, H-4028 Debrecen, Kassai u. 26. Chancellery IV, ground floor 34, website: https://unideb.hu/en/data-protection-officer-dpo, email: adatvedelmi.tisztviselo@unideb.hu, phone: +36 52,512,700/74465. The source codes are available at https://github.com/opgabor/nnUNet-vs-RECOMIA.”

E.7. Thank you for stating the following in the Competing Interests section:

The authors declare that they have no competing interests.

We note that one or more of the authors are employed by a commercial company: “Scanomed Ltd., Debrecen, Hungary”

1. Please provide an amended Funding Statement declaring this commercial affiliation, as well as a statement regarding the Role of Funders in your study. If the funding organization did not play a role in the study design, data collection and analysis, decision to publish, or preparation of the manuscript and only provided financial support in the form of authors' salaries and/or research materials, please review your statements relating to the author contributions, and ensure you have specifically and accurately indicated the role(s) that these authors had in your study. You can update author roles in the Author Contributions section of the online submission form.

Please also include the following statement within your amended Funding Statement.

“Scanomed Ltd., provided support in the form of salaries for authors [insert relevant initials], but did not have any additional role in the study design, data collection and analysis, decision to publish, or preparation of the manuscript. The specific roles of these authors are articulated in the ‘author contributions’ section.”

If your commercial affiliation did play a role in your study, please state and explain this role within your updated Funding Statement.

2. Please also provide an updated Competing Interests Statement declaring this commercial affiliation along with any other relevant declarations relating to employment, consultancy, patents, products in development, or marketed products, etc.

Within your Competing Interests Statement, please confirm that this commercial affiliation does not alter your adherence to all PLOS ONE policies on sharing data and materials by including the following statement: "This does not alter our adherence to PLOS ONE policies on sharing data and materials.” (as detailed online in our guide for authors http://journals.plos.org/plosone/s/competing-interests) . If this adherence statement is not accurate and there are restrictions on sharing of data and/or materials, please state these. Please note that we cannot proceed with consideration of your article until this information has been declared.

Please include both an updated Funding Statement and Competing Interests Statement in your cover letter. We will change the online submission form on your behalf.

• Agree – revised: Thank you for this important comment. We have stated the request for corrections and updated the Funding Statement and Competing Interests Statement in the cover letter.

• Updated Funding statement: “Project no. TKP2021-NKTA-34 has been implemented with the support provided by the Ministry of Culture and Innovation of Hungary from the National Research, Development, and Innovation Fund, financed under the TKP2021-NKTA funding scheme. The funders had no role in study design, data collection and analysis, decision to publish, or preparation of the manuscript. Scanomed Ltd., provided support in the form of salaries for authors Márton Mikó, Bence Farkas, but did not have any additional role in the study design, data collection and analysis, decision to publish, or preparation of the manuscript. The specific roles of these authors are articulated in the ‘author contributions’ section.”

• Updated Competing interest statement: “Scanomed Ltd., provided support in the form of salaries for authors Márton Mikó, Bence Farkas, but did not have any additional role in the study design, data collection and analysis, decision to publish, or preparation of the manuscript. Scanomed Ltd., affiliation does not alter our adherence to PLOS ONE policies on sharing data and materials.”

E.8. Your ethics statement should only appear in the Methods section of your manuscript. If your ethics statement is written in any section besides the Methods, please delete it from any other section.

• Agree – revised: Thank you for this important comment. Ethics statement has been deleted from the Declarations and added to “Study design and Ethical approval” point in Material and Methods.

E.9. We notice that your supplementary tables are included in the manuscript file. Please remove them and upload them with the file type 'Supporting Information'. Please ensure that each Supporting Information file has a legend listed in the manuscript after the references list.

• Agree – revised: Thank you for this important suggestion. We have removed the supplementary tables and added them to the Supporting information file. Each table in Supporting Information file has a legend listed in the manuscript after the references list.

E.10. If the reviewer comments include a recommendation to cite specific previously published works, please review and evaluate these publications to determine whether they are relevant and should be cited. There is no requirement to cite these works unless the editor has indicated otherwise.

• Agree: Thank you for this important comment.

Additional Editor Comments:

Dear Authors, please revise the paper according to the suggestions provided by the Reviewers, particularly explaining the clinical implications and usefulness of your results, and clarifying the real aim of the paper (to assess radiomic reproducibility or to find the most accurate segmentation software?). Moreover, provide more detailed discussion on radiomic parameters.

• Agree – revised: Thank you for this important comment. We have revised the manuscript according to the reviewers’ suggestions. We have added explanations about clinical implications, usefulness of the results, clarified the real aim of the paper and provided detailed discussion on radiomics parameters. We have added the OCRID of coauthors as required.

Best regards

Carmelo Caldarella

Reviewer #1: This is a paper evaluating different segmentation methods. From my point of view, it is not clear whether the aim is to assess radiomic reproducibility or to find the most accurate segmentation software. Here are my further comments:

R1.1. “accurate diagnosis requires careful examination of PET-CT images by a skilled nuclear medicine expert, which is not only tedious but also extremely time-consuming”. I do not agree, since personally I need 10-15 min for lung cancer PET reporting assessment. The above-mentioned period can be true when dealing with contrast-enhanced PET/CT or tumor segmentation or response assessment based on standardized criteria.

• Agree – revised: Thank you for your important suggestion. We agree that the original statement may not accurately reflect routine clinical PET-CT interpretation. We have revised this statement in the Introduction to according to your suggestion.

R1.2. “radiomic features offer a comprehensive characterization of the entire tumor, which is usually heterogeneous”. I would rather say that “radiomic CAN offer”

• Agree – revised: Thank you for this important suggestion. We have revised the manuscript accordingly.

R1.3. “Currently, there is a scarcity of published literature using radiomics features to compare the performance of different tumor segmentation algorithms on PET-CT images”. I hope that this is wrong since it seems that you segment tumor based on radiomic that is completely not clinically acceptable due to missing, strongly validated parameters. I think that the authors wanted to assess whether different segmentations system can obtain the same rame radiomic values.

• Agree – revised: Thank you for this important comment. It is correct that we want to assess whether different segmentations system can obtain similar radiomic values to manual reference masks. We would like to clarify that our study did not perform segmentation based on radiomics features. We have revised that sentence and added clarifications to the Introduction section to avoid confusion, according to your suggestion.

R1.4. In the whole paper, use proper radiopharmaceutical nomenclature according to EANM

• Agree – revised: Thank you for this important comment. We have revised the manuscript according to your suggestion.

R1.5. Reduce the high number of figures by moving some to supplements.

• Agree – revised: Thank you for your important suggestion. We have moved 6 figures to Supplements section.

R1.6. From the results section, it is not clear which one was the more precise segmentation compared to the manual one, as in my eyes (DSC), it is a more important parameter than the radiomic one.

• Partially Agree – revised: Thank you for this important comment. We agree that the Results section should clearly indicate which segmentation was more precise, in comparison with the manual one. We have revised the Results section to highlight the superior performance of nnU-Net.

• The novel objective of this study was to develop a complementary comparison method, based on radiomics features extracted from the segmentation outputs, which take into account the tumor shape, intensity, and texture characteristics, in addition to classical metrics when comparing segmentation algorithms. We want to highlight that Akramova and Watanabe demonstrated that radiomics is better evaluation tool than DSC for tumor segmentation performance, as it also incorporates information of tumor shape, intensity, and texture characteristics.

• Please see reference below:

o Akramova, R., & Watanabe, Y. (2024). Radiomics as a measure superior to common similarity metrics for tumor segmentation performance evaluation. Journal of applied clinical medical physics, 25(8), e14442. https://doi.org/10.1002/acm2.14442

R1.7. The results section is more similar to a table index, the authors should start presenting the main results of the whole paper.

• Agree – revised: Thank you for this important suggestion. We have revised and added the main results to the Results section.

R1.8. “This study demonstrated that nnU-Net significantly outperforms RECOMIA in terms of PET-CT solitary lung tumor segmentation, as evidenced by closer agreement of radiomic features with manual reference masks”. In my eyes, the fact that radiomic is more reproducible il much more less important than the segmentation volume DSC. This should be consider by the authors who should modify the paper structure/results presentation accordingly. Otherwise, the study would focus only on radiomic features reproducibility among different segmentation software, which, however, cannot be considered to assess a segmentation software.

• Partially Agree – revised: Thank you for this important comment. We appreciate the suggestion and we agree that conventional metrics such as DSC and HD remain essential for comparing the performance of segmentation algorithms. We would like to clarify that our goal was not to achieve reproducibility of radiomic parameters. Rather, the novel objective of this study was to develop a complementary comparison method, based on radiomics features extracted from the segmentation outputs, which take into account the tumor shape, intensity, and texture characteristics, in addition to classical metrics when comparing segmentation algorithms.

• Radiomic features agreement-based evaluation had already been used in literature for segmentation comparison. Previously, Philip et al. have applied statistical analyses (ICC, CP, TDI, CIA, LoA) on 92 radiomic features to compare performance manual and six semi-automatic segmentation, based on radiomic features agreement with manual mask. Therefore, in our view, in addition to conventional metrics such as DSC and HD, radiomics could also be used to assess performance of segmentation algorithms. We have revised the manuscript accordingly.

• Please see reference below:

o Philip, M. M., Watts, J., Moeini, S. N. M., Musheb, M., McKiddie, F., Welch, A., & Nath, M. (2024). Comparison of semi-automatic and manual segmentation methods for tumor delineation on head and neck squamous cell carcinoma (HNSCC) positron emission tomography (PET) images. Physics in medicine and biology, 69(9), 10.1088/1361-6560/ad37ea. https://doi.org/10.1088/1361-6560/ad37ea

Reviewer #2: This is a well-designed and executed study to compare two recently developed AI-based autosegmentation tools. The analysis is very comprehensive, and the results are consistent with the conclusions. I have only a few comments as shown below.

General comments

R2.1. A table summarizing the number of cases used for training and testing of two algorithms will help readers to understand the method more easily.

• Agree – revised: Thank you for this important suggestion. We have added Table 1 demonstrating the PET-CT images allocations for training and testing, together with model output.

R2.2. Radiomics features are used for performance comparison. However, the discussion of the results on radiomics is missing.

• Agree – revised: Thank you for this important comment. We have added discussion of the results on radiomics according to your suggestion.

R2.3. Furthermore, the introduction implies that radiomics application is main aim of this study by evidenced by one paragraph devoted to this topic. The first sentence of the last paragraph in Introduction gives the readers a false impression about the primary goal of this paper.

• Agree – revised: Thank you for this important comment. We have reworded that sentence to avoid misunderstanding.

R2.4. Please add more details about the radiomics feature calculations. What kind of filters were applied? Some publications show that the wavelet-based radiomics features are more sensitive to segmentation differences. See, for example, the following reference:

Akramova, R. and Y. Watanabe, Radiomics as a measure superior to common similarity metrics for tumor segmentation performance evaluation. J Appl Clin Med Phys, 2024. 25(8): p. e14442.

• Agree – revised: Thank you for this important suggestion. We have added details of radiomics features calculations accordingly.

Reviewer #3: In this paper, the authors compared the performance of two deep learning-based algorithms (mnU-Net and RECOMIA) in the segmentation of solitary lung tumor at PET images after the generation of radiomic features, these latter able to better characterize lung tumor biology.

After comparative tests, the authors conclude that radiomic features generated by mnU-Net are more similar to those derived by manual segmentation, suggesting a superiority of this segmentation algorithm.

R3.1. This manuscript is a pure methodological paper and no clinical correlation with radiomics data and features of lung cancer are provided. Which is the clinical impact of a better segmentation? Which parameters may be generated by an optimal segmentation? How it may be useful in diagnosis and monitoring of lung cancer?

• Agree – revised: Thank you for this important comment. We have added details of clinical correlation, parameters may be beneficially impacted by optimal segmentation, usefulness in diagnosis, radiotherapy planning and assessment of treatment response in the Discussion section. We have revised the limitations of this study according to your suggestion.

R3.2. Was the study retrospective or prospective? Please clarify

• Agree – revised: Thank you for this important suggestion. We have added details of study design to “Study design and Ethical approval” point in Material and Methods section.

R3.3. The projections reported in Figures 4-11 for two methods of analysis are often different. Please choose the three spatial projections for the two methods.

• Agree – revised: Thank you for this important suggestion. Because three identical spatial projections could not be obtained from RECOMIA system, to ensure harmonization, we have revised the figures showing one representative spatial projection for both methods.

Attachment Submitted filename: 20260521_Assessment_nnUNet_RECOMIA_ResponseToReviewers.pdf

# Peer review: Decision Letter 1

**DOI:** 10.1371/journal.pone.0360849.r003

**Authors:** Carmelo Caldarella

9 Jun 2026

Dear Dr. Le,

Thank you for submitting your manuscript to PLOS ONE. After careful consideration, we feel that it has merit but does not fully meet PLOS ONE’s publication criteria as it currently stands. Therefore, we invite you to submit a revised version of the manuscript that addresses the points raised during the review process.

**Main concerns;**

Please submit your revised manuscript by Jul 24 2026 11:59PM. If you will need more time than this to complete your revisions, please reply to this message or contact the journal office at plosone@plos.org. When you're ready to submit your revision, log on to https://www.editorialmanager.com/pone/ and select the 'Submissions Needing Revision' folder to locate your manuscript file.

- A letter that responds to each point raised by the academic editor and reviewer(s). You should upload this letter as a separate file labeled 'Response to Reviewers'.
- A marked-up copy of your manuscript that highlights changes made to the original version. You should upload this as a separate file labeled 'Revised Manuscript with Track Changes'.
- An unmarked version of your revised paper without tracked changes. You should upload this as a separate file labeled 'Manuscript'.

If you would like to make changes to your financial disclosure, please include your updated statement in your cover letter. Guidelines for resubmitting your figure files are available below the reviewer comments at the end of this letter.

If applicable, we recommend that you deposit your laboratory protocols in protocols.io to enhance the reproducibility of your results. Protocols.io assigns your protocol its own identifier (DOI) so that it can be cited independently in the future. For instructions see: https://journals.plos.org/plosone/s/submission-guidelines#loc-laboratory-protocols. Additionally, PLOS ONE offers an option for publishing peer-reviewed Lab Protocol articles, which describe protocols hosted on protocols.io. Read more information on sharing protocols at https://plos.org/protocols?utm_medium=editorial-email&utm_source=authorletters&utm_campaign=protocols.

As the corresponding author, your ORCID iD is verified in the submission system and will appear in the published article. PLOS supports the use of ORCID, and we encourage all coauthors to register for an ORCID iD and use it as well. Please encourage your coauthors to verify their ORCID iD within the submission system before final acceptance, as unverified ORCID iDs will not appear in the published article. *Only*  the individual author can complete the verification step; PLOS staff *cannot*  verify ORCID iDs on behalf of authors.

We look forward to receiving your revised manuscript.

Kind regards,

Carmelo Caldarella, Ph.D., M.D.

Academic Editor

PLOS One

Journal Requirements:

If the reviewer comments include a recommendation to cite specific previously published works, please review and evaluate these publications to determine whether they are relevant and should be cited. There is no requirement to cite these works unless the editor has indicated otherwise.

Additional Editor Comments:

Dear Authors, please revise the manuscript according to Reviewers' suggestions, particularly paying attention to differences between the models nnU-Net and RECOMIA (you should discuss them and, in case, demonstrate how you may detect intrinsic superiority of one segmentation algorithm over another despite such differences) and explaining why radiomics agreement should be considered a reliable indicator of segmentation performance, although radiomics are subject to high variability due to interefering parameters. Moreover, report inter- and intra-observer variability in manual segmentation to justify its use as gold standard.

Last, provide a clearer discussion of the implications of improved radiomics agreement in clinical practice.

[Note: HTML markup is below. Please do not edit.]

Reviewers' comments:

Reviewer's Responses to Questions

**Comments to the Author**

Reviewer #1: All comments have been addressed

Reviewer #4: (No Response)

**********

2. Is the manuscript technically sound, and do the data support the conclusions?

Reviewer #1: Partly

Reviewer #4: Partly

**********

3. Has the statistical analysis been performed appropriately and rigorously?

Reviewer #1: I Don't Know

Reviewer #4: No

**********

4. Have the authors made all data underlying the findings in their manuscript fully available?

The PLOS Data policy

Reviewer #1: Yes

Reviewer #4: Yes

**********

5. Is the manuscript presented in an intelligible fashion and written in standard English?

Reviewer #1: Yes

Reviewer #4: Yes

**********

Reviewer #1: (No Response)

Reviewer #4: Major Comments

1. The comparison between nnU-Net and RECOMIA is not methodologically balanced

The most important concern is that the two algorithms were evaluated under substantially different conditions.

The nnU-Net model was trained and optimized using local institutional data, scanner characteristics, acquisition protocols, and expert-approved masks derived from the same institution. In contrast, RECOMIA was evaluated as an externally pretrained model without fine-tuning on the local dataset.

Therefore, the study may primarily reflect:

domain adaptation differences,

scanner/protocol compatibility,

training data similarity,

rather than intrinsic superiority of one segmentation algorithm over another.

This issue should be discussed much more explicitly in the manuscript, and the conclusions should be substantially toned down.

2. The central assumption that “radiomics agreement reflects segmentation quality” remains insufficiently validated

The manuscript proposes radiomics feature agreement as a surrogate marker for segmentation quality. While this is an interesting concept, the biological and methodological validity of this assumption remains uncertain.

Radiomics features are highly sensitive to:

voxel spacing,

reconstruction algorithms,

smoothing,

discretization methods,

noise,

partial volume effects.

Therefore, differences in radiomics features do not necessarily indicate poorer segmentation quality.

The authors should better justify:

why radiomics agreement should be considered a reliable indicator of segmentation performance,

which radiomics features are expected to be robust,

and how non-segmentation-related variability was controlled.

Currently, the manuscript risks conflating:

radiomics reproducibility,

with

segmentation accuracy.

3. Lack of robust ground-truth validation

Manual segmentation was treated as the gold standard; however:

interobserver variability was not evaluated,

intraobserver reproducibility was not reported,

consensus procedures were insufficiently described.

This is particularly important in PET tumor delineation, where contour variability can be substantial because of:

respiratory motion,

inflammatory uptake,

partial volume effects,

threshold ambiguity.

Without demonstrating agreement between human experts, the reliability of the reference masks remains uncertain.

The manuscript would be strengthened by reporting:

manual-manual DSC,

ICC between observers,

or consensus methodology.

4. Radiomics methodology requires additional detail and justification

The radiomics analysis appears relatively simplified:

no wavelet filters,

no Laplacian-of-Gaussian features,

no harmonization strategy,

no robustness analysis,

no IBSI compliance discussion.

Furthermore, 107 radiomics features were analyzed without sufficient discussion of:

feature redundancy,

collinearity,

feature stability.

The statistical framework therefore may overestimate the significance of observed differences.

5. Statistical concerns

Several aspects of the statistical analysis require clarification:

a) Multiple testing and feature dependency

Radiomics features are highly correlated. Although Brown’s method was used, it remains unclear whether this sufficiently accounts for feature dependency.

b) Feature exclusion based on normality

The authors removed non-normally distributed features. However, radiomics features are commonly non-Gaussian. Excluding these variables may introduce bias and alter feature representation.

Please clarify:

how many features were excluded,

which feature classes were affected,

and whether sensitivity analyses were performed.

c) Interpretation of aggregate metrics

The biological and clinical interpretation of:

summed absolute correlations,

combined p-values,

remains somewhat unclear.

6. Conventional segmentation metrics are underemphasized

The manuscript places strong emphasis on radiomics agreement while relatively downplaying classical segmentation metrics such as:

Dice similarity coefficient (DSC),

Hausdorff distance (HD),

surface-based metrics.

However, in clinical practice, accurate contour delineation remains critically important, especially for:

radiotherapy planning,

response assessment,

volumetric quantification.

Radiomics agreement should probably be presented as a complementary evaluation approach rather than a replacement for established segmentation metrics.

7. Limited clinical relevance

The manuscript would benefit from a clearer discussion of the clinical implications of improved radiomics agreement.

At present, the study does not demonstrate whether higher radiomics agreement leads to improvements in:

prognosis prediction,

treatment response assessment,

radiotherapy planning accuracy,

or clinical outcomes.

Therefore, the practical clinical significance remains speculative.

8. External validation is lacking

The study is based on a single-center retrospective dataset. PET radiomics and segmentation performance are highly scanner- and protocol-dependent.

External validation using independent datasets would substantially strengthen the conclusions.

Minor Comments

Please clarify whether PET and CT channels were jointly used during segmentation inference in nnU-Net.

The manuscript would benefit from a clearer explanation of why wavelet features were not included.

Please provide additional details regarding failed segmentation cases and failure mechanisms.

Figures remain somewhat excessive despite revisions; further consolidation may improve readability.

Some wording in the Discussion still implies causal superiority of nnU-Net beyond what the data support.

**********

what does this mean?). If published, this will include your full peer review and any attached files.

If you choose “no”, your identity will remain anonymous but your review may still be made public.

**Do you want your identity to be public for this peer review?**  For information about this choice, including consent withdrawal, please see our Privacy Policy

Reviewer #1: **Yes:**  Riccardo Laudicella

Reviewer #4: No

**********

[NOTE: If reviewer comments were submitted as an attachment file, they will be attached to this email and accessible via the submission site. Please log into your account, locate the manuscript record, and check for the action link "View Attachments". If this link does not appear, there are no attachment files.]

To ensure your figures meet our technical requirements, please review our figure guidelines: https://journals.plos.org/plosone/s/figures

You may also use PLOS’s free figure tool, NAAS, to help you prepare publication quality figures: https://journals.plos.org/plosone/s/figures#loc-tools-for-figure-preparation.

NAAS will assess whether your figures meet our technical requirements by comparing each figure against our figure specifications.

# Author comment: Author response to Decision Letter 2

**DOI:** 10.1371/journal.pone.0360849.r004

17 Jul 2026

Reviewer #4:

Major Comments

R4.1. The comparison between nnU-Net and RECOMIA is not methodologically balanced

The most important concern is that the two algorithms were evaluated under substantially different conditions.

The nnU-Net model was trained and optimized using local institutional data, scanner characteristics, acquisition protocols, and expert-approved masks derived from the same institution. In contrast, RECOMIA was evaluated as an externally pretrained model without fine-tuning on the local dataset.

Therefore, the study may primarily reflect:

domain adaptation differences,

scanner/protocol compatibility,

training data similarity,

rather than intrinsic superiority of one segmentation algorithm over another.

This issue should be discussed much more explicitly in the manuscript, and the conclusions should be substantially toned down.

• Agree – revised: Thank you for your important suggestions. We have revised the Discussion section to acknowledge that the observed results may reflect domain adaptation, scanner and protocol compatibility and training data similarity. We have also toned down the conclusions accordingly.

R4.2. The central assumption that “radiomics agreement reflects segmentation quality” remains insufficiently validated

The manuscript proposes radiomics feature agreement as a surrogate marker for segmentation quality. While this is an interesting concept, the biological and methodological validity of this assumption remains uncertain.

Radiomics features are highly sensitive to:

voxel spacing,

reconstruction algorithms,

smoothing,

discretization methods,

noise,

partial volume effects.

Therefore, differences in radiomics features do not necessarily indicate poorer segmentation quality.

The authors should better justify:

why radiomics agreement should be considered a reliable indicator of segmentation performance,

which radiomics features are expected to be robust,

and how non-segmentation-related variability was controlled.

Currently, the manuscript risks conflating:

radiomics reproducibility,

with

segmentation accuracy.

• Partially agree – revised: Thank you for this important comment. We have revised the Discussion to better explain the proposed radiomics agreement-based evaluation method.

• Akramova and Watanabe demonstrated that radiomics is better evaluation tool than DSC for tumor segmentation performance. Radiomics features exhibited greater sensitivity to segmentation changes than DSC, and it also incorporates information of tumor shape, intensity, and texture characteristics.

• Regarding feature robustness, Haaburger et al demonstrated radiomics feature robustness may vary across feature classes, with shape features generally the best robust, followed by first order features. On the other hand, features quantifying shape, such as ngtdm and glcm, usually have worse reproducibility. These findings have been added to the Discussion.

• Finally, we have minimized the non-segmentation-related variability by using the identical dataset (details see Datasets section) and radiomics configuration settings to evaluate the two segmentation algorithms. Therefore, the differences in radiomics features likely indicate segmentation output quality, rather than variations in image acquisition or feature extraction protocol.

• Akramova, R. and Y. Watanabe, Radiomics as a measure superior to common similarity metrics for tumor segmentation performance evaluation. J Appl Clin Med Phys, 2024. 25(8): p. e14442 DOI: 10.1002/acm2.14442.

• Haarburger, C., et al., Radiomics feature reproducibility under inter-rater variability in segmentations of CT images. Sci Rep, 2020. 10(1): p. 12688 DOI: 10.1038/s41598-020-69534-6.

R4.3. Lack of robust ground-truth validation

Manual segmentation was treated as the gold standard; however:

interobserver variability was not evaluated,

intraobserver reproducibility was not reported,

consensus procedures were insufficiently described.

This is particularly important in PET tumor delineation, where contour variability can be substantial because of:

respiratory motion,

inflammatory uptake,

partial volume effects,

threshold ambiguity.

Without demonstrating agreement between human experts, the reliability of the reference masks remains uncertain.

The manuscript would be strengthened by reporting:

manual-manual DSC,

ICC between observers,

or consensus methodology.

• Agree – revised: Thank you for your important suggestions. We have added the interobserver and intraobserver variability analyses accordingly.

R4.4. Radiomics methodology requires additional detail and justification

The radiomics analysis appears relatively simplified:

no wavelet filters,

no Laplacian-of-Gaussian features,

no harmonization strategy,

no robustness analysis,

no IBSI compliance discussion.

Furthermore, 107 radiomics features were analyzed without sufficient discussion of:

feature redundancy,

collinearity,

feature stability.

The statistical framework therefore may overestimate the significance of observed differences.

• Partially agree – revised: Thank you for your important suggestions.

• Wavelet and Laplacian-of-Gaussian features were not included because RECOMIA only provides us with the extracted radiomics features but not the corresponding segmentation masks required to generate filtered radiomics features. To ensure a consistent comparison across all segmentation methods, the analysis was therefore restricted to the original set radiomics features that are available for all methods. We have acknowledged this in the limitation paragraph of the Discussion section.

• Harmonization strategy was not applied because all PET/CT examinations were acquired using the same scanner and protocol (details see Datasets section). Harmonization strategies are intended to reduce biases from scanner and protocol variabilities according to Mali et al. Additionally, we used the identical dataset and radiomics configuration settings to evaluate the two segmentation algorithms.

• We have added bootstrap-based robustness analysis. The ranking of segmentation algorithms remained unchanged following bootstrap analysis, which further supports the robustness of the findings.

• We have added information regarding IBSI compliance feature extraction to the Radiomics section.

• We have added discussion regarding radiomics feature redundancy, collinearity and feature stability to the Discussion section.

• Mali, S.A., et al., Making Radiomics More Reproducible across Scanner and Imaging Protocol Variations: A Review of Harmonization Methods. J Pers Med, 2021. 11(9) DOI: 10.3390/jpm11090842.

R4.5. Statistical concerns

Several aspects of the statistical analysis require clarification:

a) Multiple testing and feature dependency

Radiomics features are highly correlated. Although Brown’s method was used, it remains unclear whether this sufficiently accounts for feature dependency.

b) Feature exclusion based on normality

The authors removed non-normally distributed features. However, radiomics features are commonly non-Gaussian. Excluding these variables may introduce bias and alter feature representation.

Please clarify:

how many features were excluded,

which feature classes were affected,

and whether sensitivity analyses were performed.

c) Interpretation of aggregate metrics

The biological and clinical interpretation of:

summed absolute correlations,

combined p-values,

remains somewhat unclear.

• Agree – revised: Thank you for your important suggestions.

• a) We agree that radiomics features are highly correlated. To address this issue, we used Brown’s method specifically because it extends Fisher’s method, allow us to combine dependent p-values (Brown). However, the residual dependency of radiomics features may still affect the results. The sufficiency of this method to account for feature dependency requires further statistical analysis, which is outside of the scope of this study. We have acknowledged this in the limitation paragraph of the Discussion section.

• b) We agree that removing non-normally distributed features may introduce bias and alter feature representation. However, this is necessary based on Brown’s method multivariate normality assumption. We have acknowledged this in the limitation paragraph of the Discussion section and suggest further research using transformation method to convert non-Gaussian data to Gaussian-like shapes (for example Box Cox transformation) or other calculation methods that do not assume the normality of the data (for example Empirical Brown’s method) to generate a more reliable p-value. Details regarding excluded features are available in Table S2-S3. 99 features were excluded from the nnU-Net model 535 Brown’s method p-value calculation. 106 features were excluded from the RECOMIA Brown’s method p-value calculation. All feature classes were affected. The most affected groups are Neighboring Gray Tone Difference Matrix (NGTDM), followed by Co-occurrence Matrix (GLCM) and first order. We have performed the bootstrap analysis and added the results accordingly. Generally, the ranking of segmentation algorithms remained unchanged following bootstrap analysis, which further supports the robustness of the findings.

• c) We acknowledge that the aim of these metrics is not to provide a direct biological or clinical interpretation of individual radiomics features. However, they serve as measures of overall agreement between segmentation methods across 107 radiomic features. We have clarified this in the Discussion section.

• Brown, M.B., 400: A Method for Combining Non-Independent, One-Sided Tests of Significance. Biometrics, 1975. 31(4): p. 987-992 DOI: 10.2307/2529826.

R4.6. Conventional segmentation metrics are underemphasized

The manuscript places strong emphasis on radiomics agreement while relatively downplaying classical segmentation metrics such as:

Dice similarity coefficient (DSC),

Hausdorff distance (HD),

surface-based metrics.

However, in clinical practice, accurate contour delineation remains critically important, especially for:

radiotherapy planning,

response assessment,

volumetric quantification.

Radiomics agreement should probably be presented as a complementary evaluation approach rather than a replacement for established segmentation metrics.

• Agree – revised. Thank you for this important comment. We acknowledge that this has been mentioned in the Discussion section “Ultimately, these results suggest that radiomics features, together with our proposed comparison method may serve as valuable complementary tools to conventional metrics for evaluating and comparing segmentation algorithms in clinical and research settings.” We have also clarified this in the Conclusions section.

R4.7. Limited clinical relevance

The manuscript would benefit from a clearer discussion of the clinical implications of improved radiomics agreement.

At present, the study does not demonstrate whether higher radiomics agreement leads to improvements in:

prognosis prediction,

treatment response assessment,

radiotherapy planning accuracy,

or clinical outcomes.

Therefore, the practical clinical significance remains speculative.

• Partially agree – revised: Thank you for this important comment.

• We acknowledge that the present study was not designed to evaluate the prognosis prediction, treatment response assessment, radiotherapy planning accuracy or clinical outcomes. Rather, the objective of this study was to develop a complementary comparison method, based on radiomics features extracted from the segmentation outputs, which take into account the tumor shape, intensity, and texture characteristics, in addition to classical metrics when comparing segmentation algorithms. We have acknowledged this in the limitation paragraph of the Discussion section: “Firstly, we performed a plain methodological investigation, without any direct clinical correlation with lung cancer-related outcomes, such as diagnosis, treatment response and survival.”

• While higher radiomics agreement does not directly demonstrate improved clinical outcomes, it suggests that a segmentation method could accurately delineate the tumor, which is important for many clinical applications. Therefore, it could significantly enhance clinical outcomes and allow accurate and efficient diagnosis and treatment planning, as demonstrated by Borrelli et al and Rayed et al. We have revised the Discussion section accordingly.

• Borrelli, P., et al., Freely available convolutional neural network-based quantification of PET/CT lesions is associated with survival in patients with lung cancer. EJNMMI Phys, 2022. 9(1): p. 6 DOI: 10.1186/s40658-022-00437-3.

• Rayed, M.E., et al., Deep learning for medical image segmentation: State-of-the-art advancements and challenges. Informatics in Medicine Unlocked, 2024. 47: p. 101504 DOI: 10.1016/j.imu.2024.101504.

R4.8. External validation is lacking

The study is based on a single-center retrospective dataset. PET radiomics and segmentation performance are highly scanner- and protocol-dependent.

External validation using independent datasets would substantially strengthen the conclusions.

• Agree - revised. Thank you for this important suggestion. We agree that external validation could improve the generalizability of our findings. We have acknowledged this in the limitation paragraph of the Discussion section: “We also did not conduct external prospective validation or detailed subgroup analyses (e.g., by scanner brands, lesion size), which could reveal different context-specific performance patterns.”

Minor Comments

R4.9. Please clarify whether PET and CT channels were jointly used during segmentation inference in nnU-Net.

• Agree – revised: Thank you for this important comment. Both PET and CT images are used during training and testing by nnU-Net. We have revised the Algorithms section accordingly.

R4.10. The manuscript would benefit from a clearer explanation of why wavelet features were not included.

• Agree – revised: Thank you for this important comment. Wavelet and Laplacian-of-Gaussian features were not included because RECOMIA only provides us the extracted radiomics features but not the corresponding segmentation masks required to generate filtered radiomics features. To ensure a consistent comparison across all segmentation methods, the analysis was therefore restricted to the original set of radiomics features that are available for all methods. We have acknowledged this in the limitation paragraph of the Discussion section.

R4.11. Please provide additional details regarding failed segmentation cases and failure mechanisms.

• Agree – revised: Thank you for this important comment. We have provided additional details in the Algorithms section.

R4.12. Figures remain somewhat excessive despite revisions; further consolidation may improve readability.

• Agree – revised: Thank you for this important comment. We have consolidated Figure 4 and Figure 5 into 1 figure to improve readability.

R4.13. Some wording in the Discussion still implies causal superiority of nnU-Net beyond what the data support.

• Agree – revised: Thank you for this important comment. We have revised the Discussion and Conclusion sections accordingly.

Attachment Submitted filename: 20260717_Assessment_nnUNet_RECOMIA_ResponseToReviewers.pdf

# Peer review: Decision Letter 2

**DOI:** 10.1371/journal.pone.0360849.r005

**Authors:** Carmelo Caldarella

1 Oct 2026

Assessment of Algorithmic Performance in PET-CT Segmentation of Solitary Lung Tumors

PONE-D-26-03240R2

Dear Dr. Le,

We’re pleased to inform you that your manuscript has been judged scientifically suitable for publication and will be formally accepted for publication once it meets all outstanding technical requirements.

Within one week, you’ll receive an e-mail detailing the required amendments. When these have been addressed, you’ll receive a formal acceptance letter and your manuscript will be scheduled for publication.

An invoice will be generated when your article is formally accepted. Please note, if your institution has a publishing partnership with PLOS and your article meets the relevant criteria, all or part of your publication costs will be covered. Please make sure your user information is up-to-date by logging into Editorial Manager at Editorial Manager® and clicking the ‘Update My Information' link at the top of the page. For questions related to billing, please contact billing support.

If your institution or institutions have a press office, please notify them about your upcoming paper to help maximize its impact. If they’ll be preparing press materials, please inform our press team as soon as possible -- no later than 48 hours after receiving the formal acceptance. Your manuscript will remain under strict press embargo until 2 pm Eastern Time on the date of publication. For more information, please contact onepress@plos.org.

Kind regards,

Carmelo Caldarella, Ph.D., M.D.

Academic Editor

PLOS One

Additional Editor Comments (optional):

Reviewers' comments:

Reviewer's Responses to Questions

**Comments to the Author**

Reviewer #1: All comments have been addressed

Reviewer #3: All comments have been addressed

**********

2. Is the manuscript technically sound, and do the data support the conclusions?

Reviewer #1: Yes

Reviewer #3: (No Response)

**********

3. Has the statistical analysis been performed appropriately and rigorously?

Reviewer #1: N/A

Reviewer #3: (No Response)

**********

4. Have the authors made all data underlying the findings in their manuscript fully available?

The PLOS Data policy

Reviewer #1: Yes

Reviewer #3: (No Response)

**********

5. Is the manuscript presented in an intelligible fashion and written in standard English?

Reviewer #1: Yes

Reviewer #3: (No Response)

**********

Reviewer #1: The authors answered to the required corrections, therefore it is ready to be considered for publication

Reviewer #3: (No Response)

**********

what does this mean?). If published, this will include your full peer review and any attached files.

If you choose “no”, your identity will remain anonymous but your review may still be made public.

**Do you want your identity to be public for this peer review?**  For information about this choice, including consent withdrawal, please see our Privacy Policy

Reviewer #1: **Yes:**  Riccardo Laudicella

Reviewer #3: No

**********

# Editor report: Acceptance letter

**DOI:** 10.1371/journal.pone.0360849.r006

**Authors:** Carmelo Caldarella

PONE-D-26-03240R2

PLOS One

Dear Dr. Le,

I'm pleased to inform you that your manuscript has been deemed suitable for publication in PLOS One. Congratulations! Your manuscript is now being handed over to our production team.

At this stage, our production department will prepare your paper for publication. This includes ensuring the following:

* All references, tables, and figures are properly cited

* All relevant supporting information is included in the manuscript submission,

* There are no issues that prevent the paper from being properly typeset

You will receive further instructions from the production team, including instructions on how to review your proof when it is ready. Please keep in mind that we are working through a large volume of accepted articles, so please give us a few days to review your paper and let you know the next and final steps.

Lastly, if your institution or institutions have a press office, please let them know about your upcoming paper now to help maximize its impact. If they'll be preparing press materials, please inform our press team within the next 48 hours. Your manuscript will remain under strict press embargo until 2 pm Eastern Time on the date of publication. For more information, please contact onepress@plos.org.

You will receive an invoice from PLOS for your publication fee after your manuscript has reached the completed accept phase. If you receive an email requesting payment before acceptance or for any other service, this may be a phishing scheme. Learn how to identify phishing emails and protect your accounts at https://explore.plos.org/phishing.

If we can help with anything else, please email us at customercare@plos.org.

Thank you for submitting your work to PLOS One and supporting open access.

Kind regards,

PLOS One Editorial Office Staff

on behalf of

Dr. Carmelo Caldarella

Academic Editor

PLOS One
